# Elevated emotion network connectivity is associated with fluctuations in depression

**DOI:** 10.1073/pnas.2216499120

**Published:** 2023-10-30

**Authors:** Sean W. Kelley, Aaron J. Fisher, Chi Tak Lee, Eoghan Gallagher, Anna K. Hanlon, Ian H. Robertson, Claire M. Gillan

**Affiliations:** ^a^School of Psychology, Trinity College Dublin, Dublin D02 PN40, Ireland; ^b^Trinity College Institute of Neuroscience, Trinity College Dublin, Dublin D02 PN40, Ireland; ^c^Department of Psychology, University of California, Berkeley, CA 94720; ^d^Global Brain Health Institute, Trinity College Dublin, Dublin D02 PN40, Ireland

**Keywords:** network theory, emotions, complex systems, depression

## Abstract

Elevated emotion network connectivity is thought to leave people vulnerable to become and stay depressed. The mechanism through which this arises is however unclear. Here, we test the idea that the connectivity of emotion networks is associated with more extreme fluctuations in depression over time, rather than necessarily more severe depression. We gathered data from two independent samples of *N* = 155 paid students and *N* = 194 citizen scientists who rated their positive and negative emotions on a smartphone app twice a day and completed a weekly depression questionnaire for 8 wk. We constructed thousands of personalized emotion networks for each participant and tested whether connectivity was associated with severity of depression or its variance over 8 wk. Network connectivity was positively associated with baseline depression severity in citizen scientists, but not paid students. In contrast, 8-wk variance of depression was correlated with network connectivity in both samples. When controlling for depression variance, the association between connectivity and baseline depression severity in citizen scientists was no longer significant. We replicated these findings in an independent community sample (*N* = 519). We conclude that elevated network connectivity is associated with greater variability in depression symptoms. This variability only translates into increased severity in samples where depression is on average low and positively skewed, causing mean and variance to be more strongly correlated. These findings, although correlational, suggest that while emotional network connectivity could predispose individuals to severe depression, it could also be leveraged to bring about therapeutic improvements.

## Significance

Theories posit that emotional network connectivity confers risk for depression. This is because in connected networks, when external events trigger sadness, they activate other emotions like guilt and irritability. However, it is not clear why network connectivity is related to worsening depression, rather than having it simply become more changeable We tested this by estimating personalized emotion networks in two independent samples over 8 wk. We found strong support that network connectivity is linked to depression variance, not severity. We replicated this in a third independent community sample. This work demonstrates that emotion network connectivity captures a system’s ability to change, rather than its state at any given time, providing an understanding of how depression varies across time.

Throughout the day, our emotions are constantly changing in response to our surroundings (1, 2). Within certain bounds, these changes are regulated to be adaptive (3); in response to transient frustrations or disappointments, we feel sad temporarily, but that feeling fades, and does not necessarily impact our sense of self-worth, our sleep, or motivation. Network theories posit that psychological resilience can be captured by using a dynamical systems framework, through the study of how emotions interact with one another (4, 5). These theories predict that connected networks of emotion allow negative states to propagate through the system more easily, with negative emotions activating other negative emotions and creating positive feedback loops. A hypothesis that emerges from this conceptualization is that people with more connected emotion networks may be more prone to getting stuck in depressive states, leading to worse prognoses and poorer response to treatment (4). Preliminary evidence for these predictions has come from some small-scale studies of within-subject personalized networks, with mixed support from between-subject cross-sectional networks (6–10). However, there are conceptual problems. Network connectivity should not necessarily be related to worse symptom severity, but rather the changeability of depression over time (i.e., low resilience). According to complex systems theory, low resilience is characterized by elevated autocorrelation, variance, and cross-lagged relationships (11–13). Systems with low resilience are not able to recover from small perturbations, such as common everyday stressors, which results in an accumulation in variance in the systems’ underlying components (14). Network simulations predict this exact result; as network connectivity increases, symptoms become more variable (15). So too is this predicted by the test reliability literature in which formally, the sum of a variance–covariance matrix of items gives the variance of their total score. In the case of symptoms that make up a depression sum score, higher covariance between symptoms necessarily increases the variability of a total score (16). Recent empirical evidence for this claim found that emotion network connectivity was positively related to the absolute, but not signed, change in depression severity (17).

Why then does the current literature tend to show worsening depression linked to network connectivity? A possible explanation for these conflicting results is the reliance on cross-sectional data, which Bos et al. (18) recently showed can have striking differences in the network structure compared to within-individual networks. Where personalized networks have been studied, depression is measured infrequently and so variability cannot be quantified. But perhaps a bigger issue stems from well-established confounds between depression variance and severity that arise due to positive skew of these symptoms with substantial floor effects (19, 20).

To address this, we examined the dynamics of how emotions change throughout the day and tested the relationship with depression severity and fluctuations in depression over time. We gathered twice-daily EMA data (positive and negative affect) and weekly depression questionnaires from two samples, paid students (*N* = 155) and citizen scientists (*N* = 154), for 8 wk. We constructed personalized emotion networks for each participant and examined the association between the connectivity of those networks, depression severity, and 8-wk variability. We further tested whether these findings generalized to a large independent community sample (*N* = 519) and explored whether we could also see evidence for this in a small patient sample (*N* = 45).

## Results

### Network Connectivity and Depression.

**Fig. 1.**
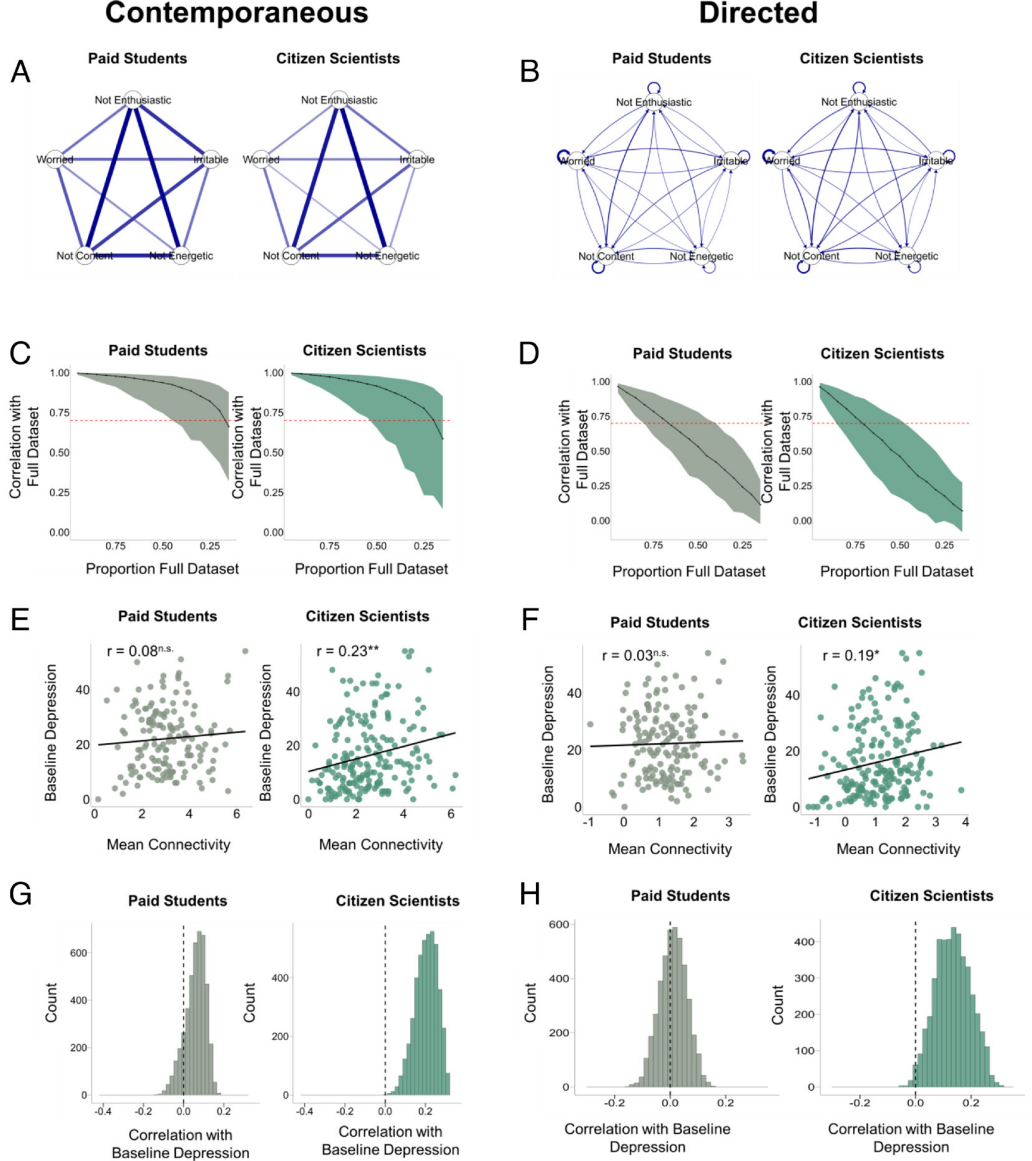
Contemporaneous and directed network connectivity in Paid Students and Citizen Scientists. (*A*) Structure of the “exemplar” contemporaneous network common to all datasets. The structure was highly consistent across samples (edges correlated at *r* = 0.97). (*B*) Exemplar directed network. The structure was highly consistent across samples (edges correlated at *r* = 0.98). (*C*) Contemporaneous networks were stable in both samples, with correlation stability values of 0.85 and 0.8 for Paid Students and Citizen Scientists respectively (where 0.5 is recommended). (*D*) Directed networks had low stability in both samples, with correlation stability values of 0.35 and 0.3 for Paid Students and Citizen Scientists, respectively. (*E*) Association of per-participant mean contemporaneous network connectivity scores and baseline depression. Citizen Scientists (*r* = 0.23), but not Paid Students (*r* = 0.08) showed a significant association with baseline depression. (*F*) Association of per-participant mean directed network connectivity scores and baseline depression. Citizen Scientists (*r* = 0.19), but not Paid Students (*r* = 0.03) showed a significant association with baseline depression. (*G*) Histograms of association between contemporaneous network connectivity and baseline depression for all possible combinations of 5-node networks in Paid Students and Citizen Scientists. (*H*) Histograms of association between directed network connectivity and baseline depression for all 5-node networks in Paid Students and Citizen Scientists. Citizen Scientists, but not Paid Students, showed a positive association between network connectivity (contemporaneous and directed) and depression.

For Paid Students and Citizen Scientists, we constructed *N* = 4,368 (i.e., all possible) 5-node networks from 16 EMA items they completed twice a day for 8 wk on the smartphone app, Neureka. A single exemplar contemporaneous network is presented in Fig. 1*A*, with edges denoting the partial correlation of emotions experienced at the same time. This network was also internally stable, with correlation stability 0.85 and 0.80 for Paid Students and Citizen Scientists, respectively (Fig. 1*C*). The structure of this network was highly consistent across the samples; edges were correlated at *r* = 0.97.

We tested for associations between contemporaneous network connectivity of each of these networks and baseline depression scores. We summarized information across these 4,368 networks to compute a per-participant average connectivity score. Individuals’ mean contemporaneous network connectivity was significantly positively associated with baseline depression in Citizen Scientists (*r* = 0.23, *P* = 0.001) but not Paid Students (*r* = 0.08, *P* = 0.34) (Fig. 1*E*). In line with the per-participant average, the resulting distribution of Pearson R correlation coefficients was nominally positive for both Paid Students (Median *r* = 0.07) and Citizen Scientists (Median *r* = 0.21) (Fig. 1*G*). However, very few 5-node networks were significant in Paid Students (proportion *P*-values < 0.05: 0.64%). In contrast, almost all Citizen Scientists’ networks showed a significant correlation, with greater connectivity linked to more severe depression symptoms (proportion *P*-values < 0.05: 86.42%). We tested the robustness of the association between connectivity and depression scores in the Citizen Scientists by testing whether connectivity was also associated with their week 8 depression rating. On the subject level, there was a significant association between the per-participant average contemporaneous network connectivity and week 8 depression in Citizen Scientists (*r* = 0.19, *P* < 0.01) but not Paid Students (*r* = −0.01, *P* = 0.89) (*SI Appendix*, Fig. S1*A*).

Moving from contemporaneous to directed networks, we found that the exemplar network was again stable across samples, with the 25 edges highly correlated *r* = 0.98 (Fig. 1*B*). In both datasets, the autoregressive coefficient of worried was the strongest edge, that is, exhibited the highest inertia. Unsurprisingly, directed networks had lower correlation stability compared to contemporaneous ones (Fig. 1*D*). Paid Students and Citizen Scientists had a CS(0.7) of 0.35 and 0.30 respectively. This is expected, however, because correlation stability means something different in the directed case, where dropping assessments does not simply reduce power, it changes the nature of the lags (degrading them from 12-h lags to 24-, 36-, 48-h lags or more). On the subject level in directed networks, we found a significant association between mean directed network connectivity and baseline depression in Citizen Scientists (*r* = 0.19, *P* = 0.01), but not Paid Students (*r* = 0.03, *P* = 0.73) (Fig. 1*F*). In permuted directed networks, Citizen Scientists (Median *r* = 0.13) and Paid Students (Median *r* = 0.01) again had nominally positive distributions of the correlations between directed network connectivity with baseline depression scores (Fig. 1*H*). Like with the contemporaneous network analysis, we found that no networks in the Paid Student sample were significantly associated with baseline depression, while 50.25% were significant in the Citizen Scientists. For directed networks, week 8 depression was also only significantly associated with connectivity in Citizen Scientists (*r* = 0.25, *P* < 0.001) but did not reach significance for Paid Students (*r* = 0.15, *P* = 0.06) (*SI Appendix*, Fig. S1*B*).

#### Depression variability.

**Fig. 2.**
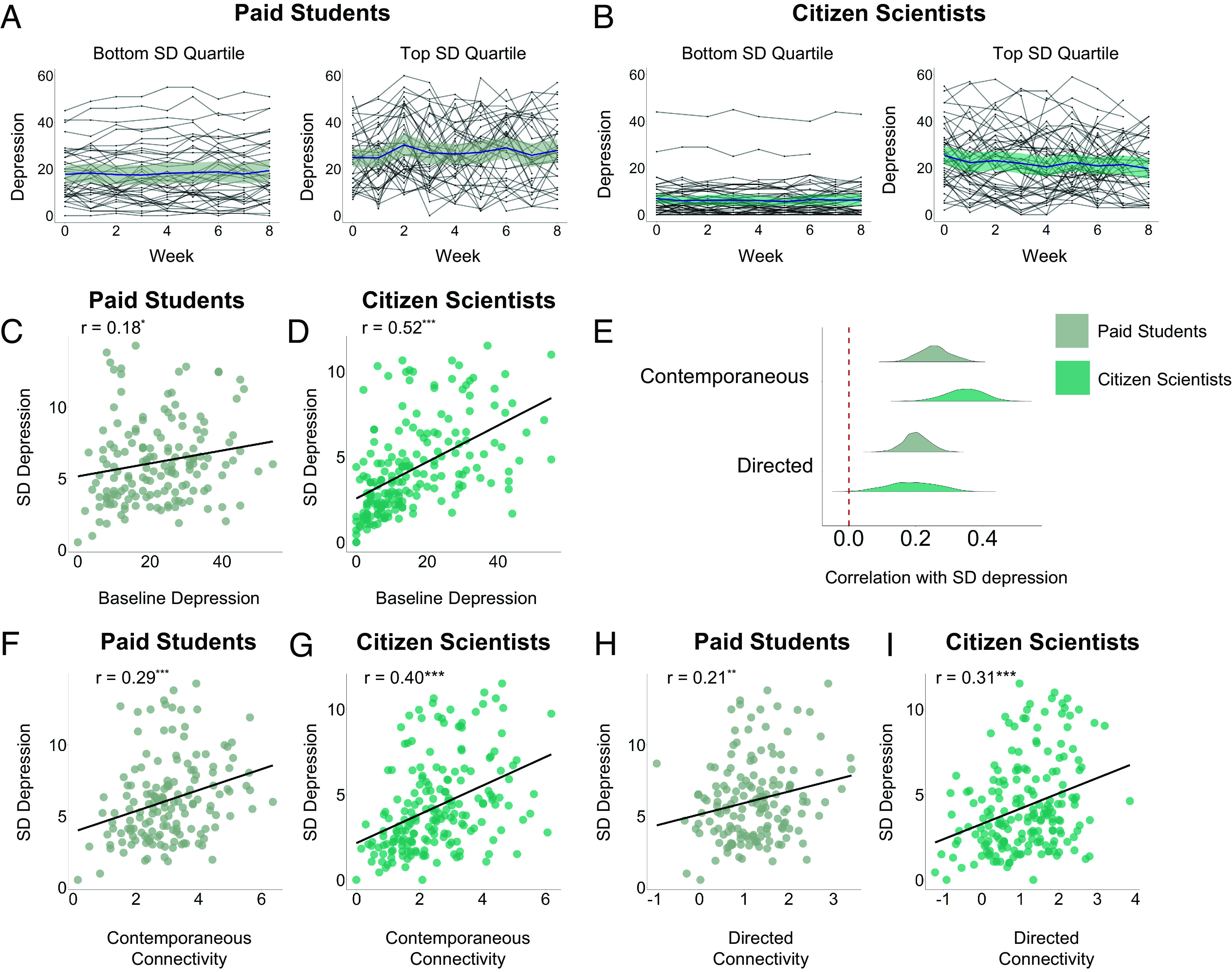
Depression variability is positively related to network connectivity. (*A*) Time series of depression scores over 8 wk of the study, split by bottom (*Left*) and top (*Right*) quantiles of SD depression for Paid Students. (*B*) Time series of depression scores over 8 wk of the study, split by bottom (*Left*) and top (*Right*) quantiles of SD depression for Citizen Scientists. (*C*) Association between baseline depression and depression variance for Paid Students. (*D*) Association between baseline depression and depression variance for Citizen Scientists. (*E*) Histograms of association between contemporaneous and directed network connectivity and 8-wk depression variability (SD) for all possible combinations of 5-node networks. (*F*) Association of per-participant mean contemporaneous network connectivity scores and depression variability for Paid Students. (*G*) Association of per-participant mean contemporaneous network connectivity scores and depression variability for Citizen Scientists. (*H*) Association of per-participant mean directed network connectivity scores and depression variability for Paid Students. (*I*) Association of per-participant mean directed network connectivity scores and depression variability for Citizen Scientists.

Moving beyond point estimates of depression severity, we assessed whether network connectivity was related to the volatility of depression over the 8-wk study period, operationalized as the SD of CES-D scores. For illustration, we plot the top and bottom quartiles from each sample (Fig. 2 *A* and *B*). In both samples, greater variability of depression was associated with higher mean depression (Paid Students: *r* = 0.18, *P* = 0.03; Citizen Scientists: *r* = 0.52, *P* < 0.001; Fig. 2 *C* and *D*), but as is clear from Fig. 2*B*, this was much more pronounced in Citizen Scientists. This finding indicates that mean and variance of depression were more confounded in Citizen Scientists compared to Paid Students. Repeating our main analysis with SD depression, we found that contemporaneous network connectivity was positively associated with SD depression in both Paid Students (Median *r* = 0.26, IQR: 0.22, 0.28; 98.53% *P*-values < 0.05) and Citizen Scientists (Median *r* = 0.35, IQR: 0.31, 0.39; 100% *P*-values < 0.05) (Fig. 2*E*). This result was also seen in the individual-level analysis, in both Paid Students (*r* = 0.29, *P* < 0.001) and Citizen Scientists (*r* = 0.40, *P* < 0.001) (Fig. 2 *F* and *G*). For directed networks, the pattern was much the same (*SI Appendix*). Directed networks were also positively associated with SD depression in Paid Students (Median *r* = 0.20, IQR: 0.18, 0.23; 36.29% *P*-values < 0.05) and Citizen Scientists (Median *r* = 0.19, IQR: 0.14, 0.24; 48.88% *P*-values < 0.05) (Fig. 2*E*). Per-participant average directed network connectivity was associated with SD Depression in both Paid Students and Citizen Scientists (*r* > 0.21, *P* > 0.008) (Fig. 2 *H* and *I*), making this the most robust clinical correlate of network connectivity identified here.

Given this, we tested whether differences in SD of depression might better explain the depression severity findings reported for the Citizen Scientists. To test this, we ran a linear regression with per-participant average connectivity as the dependent variable and baseline depression severity and SD of depression as independent variables. After controlling for SD depression, the association with baseline depression became nonsignificant in both contemporaneous (*r* = 0.23, *P* = 0.001 to *r* = −0.0004, *P* = 0.99) and directed networks (*r* = 0.19, *P* = 0.01 to *r* = 0.03, *P* = 0.69), while the SD effects remained significant in both network types (*r* > 0.10, *P* < 0.001). As a final step, we checked whether the significant association between depression variability and network connectivity is due to greater variability at the level of individual EMA items. After controlling for mean EMA item variance in Paid Students, the correlation between network connectivity and depression variability reduced to *P* = 0.05 (*r* = 0.29, *P* < 0.001 to *r* = 0.17, *P* = 0.05) (*SI Appendix*, Table S4). In Citizen Scientists, it remained significant (*r* = 0.40, *P* < 0.001 to *r* = 0.21, *P* = 0.007). Additional controls of this sort (e.g., for EMA mean, variance, and autoregression) are presented in the online supplement. (*SI Appendix*, Fig. S2).

### Experiment 2: Replication in a Large and Independent Sample (HNATD).

#### Network connectivity.

**Fig. 3.**
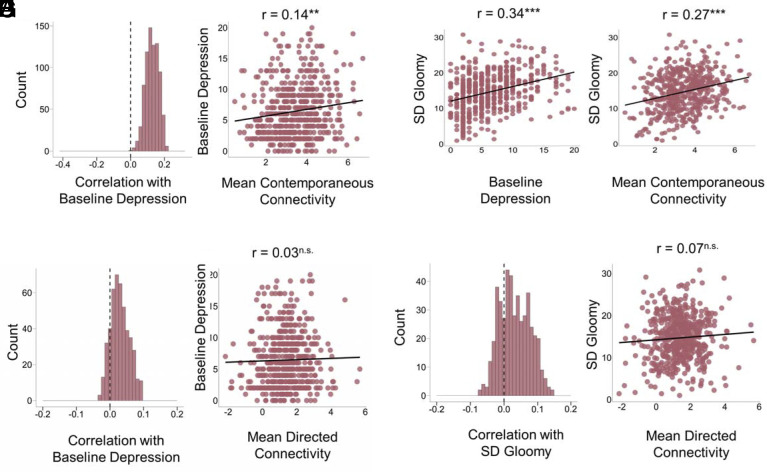
The association between network connectivity with baseline depression and SD of gloomy in a large and independent sample (HNATD). (*A*) Histograms of association between contemporaneous network connectivity and baseline depression for all possible combinations of 5-node networks in HNATD participants. (*B*) Association of per-participant mean contemporaneous network connectivity scores and baseline depression (*r* = 0.14, *P* = <0.001). (*C*) Association of baseline depression and SD gloomy (*r* = 0.34, *P* = <0.001). (*D*) Association of per-participant mean contemporaneous network connectivity scores and SD gloomy (*r* = 0.27, *P* = <0.001). (*E*) Histograms of association between directed network connectivity and baseline depression for all possible combinations of 5-node networks (*n* = 462) in HNATD participants (proportion of *P*-values < 0.05: 3.25%). (*F*) Association of per-participant mean directed network connectivity scores and baseline depression (*r* = 0.03, *P* = 0.57). (*G*) Histograms of association between directed network connectivity and SD gloomy for all possible network combinations (proportion of *P*-values < 0.05: 8.23%). (*H*) Association of per-participant mean directed network connectivity scores and baseline depression (*r* = 0.07, *P* = 0.13).

We tested whether these findings would replicate in an independent sample that used different EMA items (but included the items from the exemplar network), a different response modality (VAS 0-100 vs. Likert −3 to +3) and had a higher frequency of assessments (3 × per day), over a shorter time frame (30 d vs. 56 d). In HNATD, the exemplar contemporaneous network had a correlation stability coefficient of 0.70 (*SI Appendix*, Fig. S4*A*). Despite differences in study design, the correlation among edge strengths between HNATD and Paid Students was *r* = 0.87 and was *r* = 0.95 for Citizen Scientists. Among all combinations of 5-node networks in HNATD (*N* = 462 networks), the median correlation between contemporaneous connectivity and baseline depression severity was positive (Median *r* = 0.12) and the majority of 5-node networks were significantly associated (proportion *P*-values < 0.05: 73.81%) (Fig. 3*A*). This translated to the per-participant average connectivity analysis, where network connectivity was significantly positively associated with baseline depression (*r*(514) = 0.14, *P* = 0.002) (Fig. 3*B*). Instead of weekly depression ratings, which were not available for this sample, we used a proxy for depression variability, which we operationalized as the SD of the “I feel gloomy” EMA item—held out from all network analyses to avoid circularity. Consistent with the Paid Student and Citizen Scientist samples, the relationship between depression severity and network connectivity was explained by variance in “gloomy” over time. The SD of gloomy was positively associated with baseline depression (*r* = 0.34, *P* < 0.001) (Fig. 3*C*) and per-participant average network connectivity (*r* = 0.27, *P* < 0.001) (Fig. 3*D*). When entered in the model, it rendered the association between baseline depression and network connectivity nonsignificant (*r* = 0.14, *P* = 0.002 to *r* = 0.06, *P* = 0.20). Furthermore, the connectivity of virtually all networks (99.78% *P*-values < 0.05) was significantly associated with the SD of gloomy. Like in Citizen Scientists, the association with SD of gloomy was still significant, although reduced, after controlling for mean EMA item variance (*SI Appendix*, Table S4).

Despite being underpowered for some of the directed network analyses, considering their reduced stability (*SI Appendix*, Fig. S4*B*), we repeated all these analyses for directed networks. The exemplar directed network was also very similar to the Neureka samples, with correlations of network edge strengths of *r* = 0.87 and *r* = 0.89 between HNATD and Paid Students and Citizen Scientists, respectively. Correlation stability was higher than Neureka samples at 0.5, likely because EMA sampling was more frequent in this study (i.e., 6- vs. 12-h lags). Although slightly underpowered to examine the link between directed networks and baseline depression, we nonetheless estimated and examined these associations in an exploratory capacity. The median correlation was nominally positive, but very close to zero (Median *r* = 0.03). Just 3.25% of HNATD directed networks were significantly associated with baseline depression (Fig. 3*E*), and 8.23 % were significantly associated with SD gloomy (Fig. 3*G*). Mean directed connectivity was not significantly correlated with baseline depression (*r* = 0.03, *P* = 0.57) (Fig. 3*F*) nor SD gloomy (*r* = 0.07, *P* = 0.13) (Fig. 3*H*).

### Experiment 3: Clinical Sample.

Finally, we examined data from a much smaller clinical sample to test, in an exploratory way, if a similar pattern of results would be observed. We emphasize that this analysis is underpowered (*SI Appendix*, *Supplementary Methods*), but nevertheless informative to explore as it is conceivable that effects may be much larger in more severe patients. The structure of the contemporaneous exemplar network from the Clinical Sample was highly consistent with the three community samples (Paid Students: *r* = 0.88; Citizen Scientists: *r* = 0.96; HNATD: *r* = 0.97). This network was also stable, with a CS coefficient of 0.80 (*SI Appendix*, Fig. S5*A*). This was also the case for directed networks; correlations were high (Paid Students: *r* = 0.81; Citizen Scientists: *r* = 0.84; HNATD: *r* = 0.95) and the network had a correlation stability of 0.55 (*SI Appendix*, Fig. S5*B*).

#### Association between Network Connectivity, Baseline Depression, and Variance.

There was no association between contemporaneous [*r*(43) = −0.22, *P* = 0.15] or directed connectivity [*r*(43) = −0.04, *P* = 0.79] and baseline depression severity. The association between SD of “down or depressed” and network connectivity was also not significant for either contemporaneous (*r* = −0.005, *P* = 0.97) or directed (*r* = 0.13, *P* = 0.41) networks. Unlike the three community samples, which all showed significant relationships between variance and baseline depression severity, there was no association between the SD of the EMA item down or depressed and baseline depression (*r* = 0.11, *P* = 0.46), supporting the view that mean and variance are less confounded in datasets where depression scores are elevated and less skewed.

#### The Impact of Node Valence.

We tested whether the number of negative emotions in a network would increase the association between network connectivity and depression variance over the course of the study in those samples where significant effects were observed. For contemporaneous networks, we found positive associations in Paid Students, *r* = 0.67, *P* < 0.001, Citizen Scientists, *r* = 0.16, *p* < 0.001 and HNATD (for SD gloomy), *r* = 0.25, *p* > 0.001 (*SI Appendix*, Fig. S6*A*). For directed networks, the results were similar, albeit somewhat smaller, networks with more negative nodes had stronger associations to depression variance (Paid Students, *r* = 0.06; Citizen Scientists, *r* = 0.17; HNATD, *r* = 0.27) (*SI Appendix*, Fig. S6*B*).

#### Dynamic Temporality of Effects.

It is a limitation of the study design that network estimates and depression scores are estimated over the same 8-wk period. To partially address this, and probe the temporal dependence of these effects, we conducted a final exploratory analysis in the Neureka samples. Specifically, we generated weekly averages of contemporaneous network connectivity and tested if weekly depression symptoms were significantly associated with connectivity metrics estimated the week prior to, or following, that assessment. We found evidence for the former. In Paid Students, connectivity measures predicted future depression scores, *β* = 0.59 (SE = 0.17), *t* = 3.44, *P* < 0.011, but did not follow them *β* = −0.09 (SE = 0.17), *t* = −0.54, *P* = 0.59 (Table 1). The same was observed for citizen scientists; connectivity the week before predicted depression scores, *β* = 0.59 (SE = 0.13), *t* = 4.75, *P* < 0.001, but did not follow them *β* = −0.06 (SE = 0.13), *t* = 0.47, *P* = 0.64.

**Table 1.** Weekly lagged relationships between contemporaneous connectivity and depression scores

DV	Dataset	IV	Beta (SE)	*t*	*P*-value
Depression	Paid Students	Connectivity (before)	0.59 (0.17)	3.44	<0.001
		Connectivity (after)	−0.09 (0.17)	−0.54	0.59
	Citizen Scientists	Connectivity (before)	0.59 (0.13)	4.75	<0.001
		Connectivity (after)	0.06 (0.13)	0.47	0.64

Independent variables (IVs) of contemporaneous network connectivity estimated from the week “before” or “after” the dependent variable (DV) of depression were gathered.

## Discussion

Network theory posits that depression is the result of causal interactions among disorder-relevant emotional experiences like sadness, guilt, or a lack of motivation. One of its key predictions is that people with more connected networks are less resilient, and therefore more vulnerable to depression. However, the studies to date that have tried to test this assumption have had small samples or too few assessments per person to reliably estimate individual differences in personalized network connectivity. Moreover, although models exist that describe how connectivity of networks of binary symptom data translates into psychiatric vulnerability (4), the mechanism through which this process occurs for emotional states that are experienced by degree, has not been fleshed out. Here we tested the idea that associations between network connectivity and increased depression severity, when present, are explained by the variability of depression over time.

We estimated personalized network connectivity in two large samples who completed twice daily EMA assessments of positive and negative affect, alongside weekly standardized depression questionnaires for 8 wk. Baseline depression severity was related to contemporaneous network connectivity in Citizen Scientists, but not Paid Students. In contrast, 8-wk depression variability was related to network connectivity in both samples. After we controlled for depression variability, the association between contemporaneous connectivity and depression became nonsignificant. These findings were replicated in a third large independent community sample. Results were largely consistent for directed networks, though effects were smaller due to reduced stability. Across all datasets and network types, results were stronger when networks contained more negative affect items. We conclude that network connectivity is related primarily to fluctuations in depression over time, and in some samples (but not all), this manifests in higher severity. One way to think of this conclusion is through a resilience framework. Truly elevated network connectivity means that changing one symptom is likely to cause downstream, coherent, changes in others. But sparsely connected networks are less likely to result in large variation in depression severity and thus can be viewed as more resilient; however, depending on the set-point of depression, this “resilience” can be for better or worse. In line with this, unstable emotional dynamics have been linked to better treatment outcomes in mood disorders (21, 22), obsessive-compulsive disorder (23, 24), childhood aggression (25), and personality disorders (26). In people with high levels of negative affect, greater negative affect variability is associated with fewer depressive symptoms (27). This implies that when negative emotions are already high, greater variability indicates the system can change in a positive direction. Another consistent finding in the cross-sectional network literature is that depression network connectivity increases, rather than decreases, after successful treatment (28–31). Likewise, among patients diagnosed with a psychotic disorder, positive and negative symptom networks increase in connectivity when patients respond to treatment (32).

Although there can be benefits, studies (including the present study) have shown that systems with low resilience are in some settings associated with worse outcomes and deterioration. Individuals with low trait resilience have stronger associations between positive and negative emotions, especially when stressed (33). Adolescents who show a slower recovery in their negative affect—which is a sign of low resilience—after an unpleasant event, tended to also experience elevated depression severity (34). Longitudinal n-of-1 studies in people with depression found increasing variance of mental health states prior to a significant increase in depression severity (35, 36). Collectively, these results are consistent with the idea that network connectivity predisposes people to have more variable symptoms, without a particular directional change. Within psychotherapy, elevated emotional instability could enable the integration of information, reduce the entrenchment of the current pathological state, and facilitate the transition into a healthy state. Network connectivity may be a promising marker of treatment response and a way to understand the mechanism of action.

Mathematically, the results presented here may reflect a property of the variance of depression sum scores—the variance of any sum score is equal to the sum of the variance-covariance matrix of its constituent items (16). Network connectivity in the present study comes from a VAR model and thus reflects the covariance between emotional states—not items—from the CES-D; insofar as momentary positive and negative affect can be considered similar to core symptoms of depression, it follows that when covariance of these emotions is higher, so too is the variance-covariance matrix of those same items, and therefore the overall variance of their sum. This raises an important distinction between binary symptom models (e.g., the Ising model) upon which network theory was initially framed (37) and the ordinal one applied here (e.g., VAR), which make different predictions about how connectivity of items relate to changes in the total score (4). Future research should directly compare binary versus ordinal models of depression symptoms to understand which conceptualization best explains how depression unfolds over time.

It is important to acknowledge the limitations of our study. First and foremost, the design of this study is correlational. Although network theory is based firmly on the premise that symptoms have causal relations to one another, studies that are observational in nature cannot test this. By examining lagged effects in our directed networks, we can test necessary conditions for causation such as temporal precedence, but we cannot exclude the possibility that third variables (e.g., environmental events) explain the associations we observed. Another limitation is that all of our primary analyses concern data gathered over the same 8-wk period. To partially address this, we examined lagged weekly effects of connectivity on depression and found evidence that connectivity precedes changes in depression, not the other way around. Although suggestive, future work should more fully separate the time series of network connectivity and depression assessments. Personalized networks were estimated during vector autoregression, which assumes that emotion dynamics are stationary, i.e., constant mean and variance over time. This assumption may be reasonable in relatively healthy participants but would not hold in people undergoing treatment for depression. It is possible that network connectivity could substantially change with the addition of other emotions. However, estimating all possible combinations of networks and using external data with a slightly different EMA composition helps to ameliorate this concern. Another open question concerns the appropriate sampling rate for directed network estimation. Emotional states themselves have different durations (38), and it is unclear how we should sample emotions relative to those intrinsic timescales. To partially address this, we can look at the HNATD dataset, which had 6-h intervals, compared to the 12-h intervals of the main Neureka dataset. In HNATD, we found much weaker relationships between directed connectivity and depression mean and fluctuations over 8 wk, which may suggest that 6-h intervals are too fast. In our replication dataset, we did not have access to a repeated and longitudinal assessment of depression using a validated scale; instead, we constructed a proxy for depression variance from the EMA item I feel gloomy. This therefore serves as an indicative or conceptual replication of the results of the two samples in experiment 1.

We found evidence that network connectivity is associated with symptom severity in some settings, but that this effect can be explained by depression variability. Given that recent studies produced mixed results regarding the role of network connectivity in mental health, shifting focus from severity to variance may resolve these inconsistencies. Network theory offers a powerful framework for explaining the development, maintenance, and evolution of mental health disorders. Future tests of this theory should move from correlational, observational studies to causal interventional designs.

## Methods and Materials

### Experiment 1: Paid Students and Citizen Scientists in Neureka.

#### Participants.

We recruited EMA data via a smartphone app, Neureka, from two independent samples. The first were “Paid Students” gathered in multiple waves between September 2019 and April 2021, currently enrolled at universities in the Republic of Ireland. The second sample comprised members of the public who downloaded the Neureka App from the app store and participated on a voluntary basis between June 2020 and June 2022. They are here on referred to as “Citizen Scientists.” After applying the exclusion criteria (detailed below), data from *N* = 155 paid students and *N* = 194 citizen scientists were analyzed. Students in the final sample had a mean age of 23 y (SD: 4.7 y, range: 18–41), 74.8% of the sample was female, and 54.2% of participants were depressed. Citizen scientists were older, less female, and less depressed: mean age of 49.5 y (SD: 13.6 y, range: 18–82), 64.9% female, and 32.5% depressed (*SI Appendix*, Table S1). All experiments reported here received approval from the School of Psychology at Trinity College Dublin and all participants provided informed consent.

#### Procedure.

Participants registered for “Multi-Mood,” an 8-wk ecological momentary assessment study within Neureka. On sign-up, users completed the Center for Epidemiologic Studies–Depression Scale (CES-D 20) and selected two 3-h time intervals per day, between 6:00–11:30 am and 6:00–11:30 pm, when they would like to receive twice daily notifications to rate their mood. This resulted in equidistant assessments, 2 per day, spaced 12 h apart. The notification appeared randomly within that 3-h interval and participants could not rate their mood until the notification appeared. After receiving the notification, participants had until the end of the 3-h window to complete their assessment. Participants rated their mood on 9 negative and 7 positive affect items, e.g., “I feel down” (*SI Appendix*, Table S2). Items were rated on a 7-point Likert scale from -3 (not at all) to +3 (very much so). Once a week, participants repeated the CES-D 20 (39).

Of the 277 participants recruited for the student arm, 249 completed at least 1 assessment, and 164 completed at least 75% of assessments, which was the threshold for inclusion in data analysis applied to all datasets described in this paper. A further 9 participants were excluded for not completing at least 7 out of 9 weekly depression questionnaires, leaving data from a final N of 155 in this arm, with their characteristics displayed in *SI Appendix*, Table S1.

In the citizen scientist arm, 3,854 participants signed up to Multi-Mood with 1,739 completing at least 1 assessment and 222 completing at least 75% of assessments. Of the participants who completed at least 75% of assessments, 28 were excluded for not completing at least 7 of 9 weekly depression questionnaires. After these exclusions were applied, data from a final sample of 194 Citizen Scientists were analyzed (*SI Appendix*, Table S1). Further details regarding data preparation steps are available in *SI Appendix*, *Supplementary Methods*.

#### Network estimation.

Time series of EMA items were used to construct personalized directed and contemporaneous networks for each participant. Directed networks allow us to understand temporal dependence between emotions via time-lagged associations. Contemporaneous networks summarize the undirected associations between emotions, at the same moment in time. In directed and contemporaneous networks, all EMA items are considered “nodes.” The “edges” between these nodes refer to their directed or contemporaneous (partial) correlation. To construct these networks, we used a vector-autoregressive (VAR) model using the vars package in R. In a VAR model, the dependent variable is the time series of the EMA item and the independent variables are the lagged (*t*−1) version of this item’s time series, plus the lagged time series of all other EMA items. Analyses described in this paper are based on 5-node networks, that are based on 5 time-lagged regression models. An example of one of these regressions is as follows: ${\mathrm{Worried}}_{t}=\beta_{1}{\mathrm{Worried}}_{t-1}+\beta_{2}Not\;{\mathrm{Enthusiastic}}_{t-1}+\;\beta_{3}Not\;{\mathrm{Energetic}}_{t-1}+$ $\beta_{4}{\mathrm{Irritable}}_{t-1}+\;\beta_{5}{Not\;Content}_{t-1}$. From this analysis, we can determine the autocorrelation of “worried” onto itself ( $\beta_{1}$ ), as well as the “influence” of the other 4 nodes onto worried ( $\beta_{2},\beta_{3},\beta_{4},\beta_{5}).$ Repeating this regression with the other 4 nodes as the DV results in 25 regression coefficients which represent the edges of the directed network. The contemporaneous network is constructed from the correlation of residuals from these regressions. In both kinds of networks, overall network connectivity was operationalized as the sum of all (signed) edges. Unlike studies interested in the structure of individual networks, we did not implement any penalization to the edges (e.g., setting edges to zero if their weight was uncertain). Because raw networks were aggregated (e.g., to get a mean connectivity score), we wanted to avoid any loss of information due to penalization.

#### Network stability and generalizability.

To test for the stability and generalizability of the network structure, we focused on a single network (directed and contemporaneous) of 5-nodes that were common to each dataset (including in experiments 2 and 3). These were worried, “irritable,” “not enthusiastic,” “not content,” and “not energetic.” Within each study, we visualized the mean network structure of this exemplar network by averaging each edge across participants to produce an average for each dataset. We tested whether network structures were stable using a network stability test (*SI Appendix*, *Supplementary Methods*).

#### Permuted network analysis.

Although we gathered data on 16 EMA items, prior research showed that networks constructed from large sets of items are less stable than smaller networks (40). We considered engaging in both theory and data-driven selection to reduce the total number of EMA items. But without any strong a priori conviction, we considered all possible networks and focused on meta-results that would be more robust and generalizable. We therefore estimated all 4,368 possible 5-node network compositions for every participant in the two samples. For each individual network, we calculated the Pearson’s R correlation between network connectivity and baseline depression. This gave us an item-agnostic view of how all possible emotion networks behave, ensuring results were not driven by a single 5-node selection. We removed any outliers in network connectivity and depression greater than or equal to 3 SDs from the mean on a per-analysis basis. To summarize the overall pattern of association, we plotted the distribution of these correlation coefficients.

#### Per-participant average connectivity.

We took the average of connectivity of these 4,368 networks for each person in the study, producing their “per-person average connectivity” score. This value was then used as an individual difference measure that we correlated with depression. It was also used to determine the sample size needed to replicate the effects in external data (*SI Appendix*, *Results*).

#### Week 8 depression and depression variance.

We tested whether our baseline results generalized to depression scores reported at the end of the study. We tested whether variance of depression (SD over 8 wk) was a stronger correlate of network connectivity than these point estimates of depression severity.

### Experiment 2: Replication in a Large and Independent Citizen Science Sample (HNATD).

#### Participants.

To replicate these findings externally, we applied our analyses to the “How Nuts Are The Dutch” (HNATD) dataset (41). HNATD was a crowdsourced EMA study conducted in the Netherlands that recruited over 12,000 general participants between May 2014 and December 2018. We extracted complete datasets from *N* = 519 participants who were unpaid and had an average age in the 40 s (*M* = 40.3 y (SD: 13.6, range: 17–73) (*SI Appendix*, Table S1). Participants were more female than the Citizen Scientists (83%) and with a higher proportion classified as depressed based on their self-report scores (51.3%; though note different depression instruments have different sensitivities and specificities in their thresholds). HNATD participants completed three assessments per day for 30 d with assessments spaced out at 6-h intervals. Further details are available in *SI Appendix*, *Supplementary Methods*.

### Experiment 3: Comparison of Clinical and Nonclinical Network Structure and Stability.

#### Participants.

The final sample included was a “Clinical Sample” diagnosed with anxiety or depression (42). We classed this analysis as exploratory, as the power analysis from experiment 1 suggests that at *N* = 45, it is underpowered to estimate the effects observed in our nonclinical samples. Nonetheless, we felt that it might be informative for future studies to apply our analysis to a Clinical Sample (95% depressed; *SI Appendix*, Table S1).

#### Cross-experiment comparisons.

To assess consistency across our different samples, we constructed a single exemplar network (directed and contemporaneous) of 5 EMA items that were present in each dataset (worried, irritable, not enthusiastic, not content, and not energetic). Within each study, we summarized and visualized the mean network structure of this exemplar network by averaging each edge across participants. We tested whether network structures were reliable using a network stability test and cross-sample correlations of edge strengths (*SI Appendix*, *Supplementary Methods*).

## Supplementary Material

Appendix 01 (PDF)

## Data Availability

Data from experiment 1 and all analysis codes (experiments 1, 2, and 3) are available at https://osf.io/wz6rn/ (43). Data from experiment 2 are available at https://osf.io/5ybxt/ (44). Data from experiment 3 are available upon request from the original authors. Previously published data were used for this work (41, 42).

## References

[1] C. S. Carver, Control processes, priority management, and affective dynamics. Emotion Rev. **7**, 301–307 (2015).

[2] J. T. Cacioppo, W. L. Gardner, Emotion. Annu. Rev. Psychol. **50**, 191–214 (1999). 10074678 10.1146/annurev.psych.50.1.191

[3] T. B. Kashdan, J. Rottenberg, Psychological flexibility as a fundamental aspect of health. Clin. Psychol. Rev. **30**, 865–878 (2010). 21151705 10.1016/j.cpr.2010.03.001 PMC2998793

[4] A. O. Cramer , Major depression as a complex dynamic system. PLoS ONE **11**, e0167490 (2016). 27930698 10.1371/journal.pone.0167490 PMC5145163

[5] D. Borsboom, A. O. Cramer, Network analysis: An integrative approach to the structure of psychopathology. Annu. Rev. Clin. Psychol. **9**, 91–121 (2013). 23537483 10.1146/annurev-clinpsy-050212-185608

[6] M. Lee Pe , Emotion-network density in major depressive disorder. Clin. Psychol. Sci. **3**, 292–300 (2015). 31754552 10.1177/2167702614540645 PMC6871506

[7] J. T. Wigman , Exploring the underlying structure of mental disorders: Cross-diagnostic differences and similarities from a network perspective using both a top-down and a bottom-up approach. Psychol. Med. **45**, 2375–2387 (2015). 25804221 10.1017/S0033291715000331

[8] D. M. Lydon-Staley , Adolescent emotion network dynamics in daily life and implications for depression. J. Abnormal Child Psychol. **47**, 717–729 (2019). 10.1007/s10802-018-0474-y PMC6411453 30203118

[9] K. E. Shin, M. G. Newman, N. C. Jacobson, Emotion network density is a potential clinical marker for anxiety and depression: Comparison of ecological momentary assessment and daily diary. Br. J. Clin. Psychol. **61**, 31–50 (2022). 33963538 10.1111/bjc.12295 PMC8572316

[10] I. A. van de Leemput , Critical slowing down as early warning for the onset and termination of depression. Proc. Natl. Acad. Sci. U.S.A. **111**, 87–92 (2014). 24324144 10.1073/pnas.1312114110 PMC3890822

[11] M. Scheffer , Early-warning signals for critical transitions. Nature **461**, 53–59 (2009). 19727193 10.1038/nature08227

[12] V. Dakos , Spatial correlation as leading indicator of catastrophic shifts. Theor. Ecol. **3**, 163–174 (2010).

[13] S. R. Carpenter, W. A. Brock, Rising variance: A leading indicator of ecological transition. Ecol. Lett. **9**, 311–318 (2006). 16958897 10.1111/j.1461-0248.2005.00877.x

[14] L. Chen , Detecting early-warning signals for sudden deterioration of complex diseases by dynamical network biomarkers. Sci. Rep. **2**, 342 (2012). 22461973 10.1038/srep00342 PMC3314989

[15] G. Lunansky , The mental health ecosystem: extending symptom networks with risk and protective factors. Front. Psychiatry **12**, 640658 (2021). 33815173 10.3389/fpsyt.2021.640658 PMC8012560

[16] L. J. Cronbach, W. G. Warrington, Time-limit tests: Estimating their reliability and degree of speeding. Psychometrika **16**, 167–188 (1951). 14844557 10.1007/BF02289113

[17] G. Lunansky, R. H. Hoekstra, T. Blanken, Disentangling dynamic affect trajectories for distinct depression courses during the COVID-19 pandemic. PsyArXiv [Preprint] (2021). 10.31234/osf.io/hv4cb (Accessed 4 August 2023).

[18] F. M. Bos , Can we jump from cross-sectional to dynamic interpretations of networks? Implications for the network perspective in psychiatry. Psychother. Psychosom. **86**, 175–177 (2017). 28490028 10.1159/000453583 PMC5516409

[19] W. R. Ringwald, A. G. Wright, Overcoming the confound of means and variability for measuring everyday emotion dynamics related to neuroticism. PsyArXiv [Preprint] (2022). 10.31234/osf.io/nxbyd (Accessed 8 July 2023).

[20] E. Dejonckheere , Complex affect dynamics add limited information to the prediction of psychological well-being. Nat. Hum. Behav. **3**, 478–491 (2019). 30988484 10.1038/s41562-019-0555-0

[21] M. Olthof , Destabilization in self-ratings of the psychotherapeutic process is associated with better treatment outcome in patients with mood disorders. Psychother. Res. **30**, 520–531 (2020). 31256713 10.1080/10503307.2019.1633484

[22] A. M. Hayes, J. L. Strauss, Dynamic systems theory as a paradigm for the study of change in psychotherapy: An application to cognitive therapy for depression. J. Consult. Clin. Psychol. **66**, 939 (1998). 9874907 10.1037//0022-006x.66.6.939

[23] G. K. Schiepek, I. Tominschek, S. Heinzel, Self-organization in psychotherapy: Testing the synergetic model of change processes. Front. Psychol. **5**, 1089 (2014). 25324801 10.3389/fpsyg.2014.01089 PMC4183104

[24] S. Heinzel, I. Tominschek, G. Schiepek, Dynamic patterns in psychotherapy-discontinuous changes and critical instabilities during the treatment of obsessive compulsive disorder. Nonlinear Dyn. Psychol. Life Sci. **18**, 155–176 (2014). 24560009

[25] A. Lichtwarck-Aschoff , A characteristic destabilization profile in parent-child interactions associated with treatment efficacy for aggressive children. Nonlinear Dyn. Psychol. Life Sci. **16**, 353 (2012). 22695153

[26] A. M. Hayes, C. Yasinski, Pattern destabilization and emotional processing in cognitive therapy for personality disorders. Front. Psychol. **6**, 107 (2015). 25755647 10.3389/fpsyg.2015.00107 PMC4337234

[27] D. Maciejewski , Beyond main effects? Affect level as a moderator in the relation between affect dynamics and depressive symptoms. PsyArXiv [Preprint] (2022). 10.31234/osf.io/42uzq (Accessed 23 June 2023).

[28] C. Beard , Network analysis of depression and anxiety symptom relationships in a psychiatric sample. Psychol. Med. **46**, 3359–3369 (2016). 27623748 10.1017/S0033291716002300 PMC5430082

[29] M. T. Berlim , The network structure of core depressive symptom-domains in major depressive disorder following antidepressant treatment: A randomized clinical trial. Psychol. Med. **51**, 2399–2413 (2021). 32312344 10.1017/S0033291720001002

[30] F. M. Bos , Cross-sectional networks of depressive symptoms before and after antidepressant medication treatment. Soc. Psychiatry Psychiatr. Epidemiol. **53**, 617–627 (2018). 29627898 10.1007/s00127-018-1506-1 PMC5959987

[31] I. Höller , The mereology of depression—Networks of depressive symptoms during the course of psychotherapy. Int. J. Environ. Res. Public Health **19**, 7131 (2022). 35742380 10.3390/ijerph19127131 PMC9222343

[32] F. Z. Esfahlani , Sensitivity of the positive and negative syndrome scale (PANSS) in detecting treatment effects via network analysis. Innov. Clin. Neurosci. **14**, 59 (2017). 29410938 PMC5788252

[33] A. D. Ong , Psychological resilience, positive emotions, and successful adaptation to stress in later life. J. Person. Soc. Psychol. **91**, 730 (2006). 10.1037/0022-3514.91.4.730 17014296

[34] A. Kuranova , Measuring resilience prospectively as the speed of affect recovery in daily life: A complex systems perspective on mental health. BMC Med. **18**, 1–11 (2020). 32066437 10.1186/s12916-020-1500-9 PMC7027206

[35] M. Wichers , Critical slowing down as a personalized early warning signal for depression. Psychother. Psychosom. **85**, 114–116 (2016). 26821231 10.1159/000441458

[36] M. Wichers, A. C. Smit, E. Snippe, Early warning signals based on momentary affect dynamics can expose nearby transitions in depression: A confirmatory single-subject time-series study. J. Person-Oriented Res. **6**, 1 (2020). 10.17505/jpor.2020.22042 PMC7842626 33569148

[37] D. Borsboom, A network theory of mental disorders. World Psychiatry **16**, 5–13 (2017). 28127906 10.1002/wps.20375 PMC5269502

[38] P. Verduyn, S. Lavrijsen, Which emotions last longest and why: The role of event importance and rumination. Motiv. Emotion **39**, 119–127 (2015).

[39] E. M. Andresen , Screening for depression in well older adults: Evaluation of a short form of the CES-D. Am. J. Prevent. Med. **10**, 77–84 (1994). 8037935

[40] A. C. Mansueto , Investigating the feasibility of idiographic network models. Psychol. Methods **28**, 1052–1068 (2023). 34990189 10.1037/met0000466

[41] L. V. D. Krieke , HowNutsAreTheDutch (HoeGekIsNL): A crowdsourcing study of mental symptoms and strengths. Int. J. Methods Psychiatric Res. **25**, 123–144 (2016). 10.1002/mpr.1495 PMC6877205 26395198

[42] A. J. Fisher , Exploring the idiographic dynamics of mood and anxiety via network analysis. J. Abnormal Psychol. **126**, 1044 (2017). 10.1037/abn0000311 29154565

[43] S. W. Kelley , Elevated Emotion Network Connectivity Leaves People Vulnerable to Fluctuations in Depression. OSF. https://osf.io/wz6rn/. Deposited 25 September 2022.

[44] A. J. Fisher, J. W. Reeves, G. Lawyer, J. D. Medaglia, J. A. Rubel, Exploring the Idiographic Dynamics of Mood and Anxiety via Network Analysis. OSF. https://osf.io/5ybxt/. Deposited 28 July 2017. 10.1037/abn0000311 29154565

