# Supplementary material for: Elevated emotion network connectivity is associated with fluctuations in depression
Source: Proc Natl Acad Sci U S A. 2023 Oct 30;120(45):e2216499120. doi: 10.1073/pnas.2216499120 (PMC10636367; doi:10.1073/pnas.2216499120)
Supplement: Supplementary file 1 — Appendix 01 (PDF) [file pnas.2216499120.sapp.pdf]

Supporting Information for

Elevated Emotion Network Connectivity is Associated With Fluctuations in Depression

Sean W Kelley[1,2], Aaron Fisher[3], Chi Tak Lee[1,2], Eoghan Gallagher[1,2], Anna K Hanlon[1,2], Ian H Robertson[2,4] and Claire M Gillan[1,2,4*]

[1]School of Psychology, Trinity College Dublin, Dublin, Ireland
[2]Trinity College Institute of Neuroscience, Trinity College Dublin, Dublin, Ireland
[3]Department of Psychology, University of California, Berkeley
[4]Global Brain Health Institute, Trinity College Dublin, Dublin, Ireland

*Corresponding Author: Sean W Kelley

Email: seanwk21@gmail.com

This PDF file includes:

Supplementary Text
Figures S1 – S8
Tables S1 – S6
SI References

METHODS FOR EXPERIMENT 1: PAID STUDENTS AND CITIZEN SCIENTISTS IN NEUREKA 4

Procedure. 4
Data Preparation. 4
Sensitivity Analyses. 4
Permutation Tests. 5
Network Stability Test. 5
Observed Power from Experiment 1. **6**
Methods for Experiment 2: Replication in a large and independent Citizen Science sample (HNATD) **6**
Procedure. 7
Data Preparation. 7
Data Analysis. 7
Methods for Experiment 3: Comparison of Clinical and Non-Clinical Network Structure and Stability **8**
Procedure. 8
Data Preparation. 8
Data Analysis. 8
Table S1: Comparison of demographic characteristics between Paid Students, Citizen Scientists, HNATD, and a clinical sample. **9**
Table S2: Positive and negative affect items used to construct 5-node networks **10**

SUPPLEMENTARY RESULTS **11**

Control and Sensitivity Analyses in Experiment 1. **11**
Table S3: Association between network connectivity in the exemplar 5-node network with SD depression (Paid Students & Citizen Scientists), SD gloomy (HNATD), and SD down or depressed (Clinical Sample). **12**
Figure S1: Association of per-participant network connectivity and Week-8 depression. **14**
Figure S2: Comparative analysis of strength of association (x-axis, beta) between network connectivity and 8-week depression variance and other metrics derived from EMA (Mean, Standard Deviation [SD] and autoregression [AR]). **15**
Figure S3. Replication of results using 'mean depression' instead of baseline depression. **15**
Table S4: Association between depression variability and network connectivity either unadjusted or adjusted for mean EMA item variance. **16**
Table S5: Results from full 16-node network are similar to the average of the 4,368 5-node networks. **16**
Table S6: Proportion of variance explained for models predicting depression variance in Paid Students and Citizen Scientists. **17**
Figure S4: Exemplar network stability in a large independent sample (HNATD). **18**
Figure S5: Exemplar network stability in a clinical sample **19**
Figure S6: Impact of node valence on relationship between network connectivity and depression variability **20**
Figure S7. Regularised Contemporaneous and Directed Network Connectivity in Paid Students and Citizen Scientists. **21**
Figure S8. Depression variability is positively related to regularised network connectivity **22**

SUPPLEMENTARY REFERENCES 22

Supplementary Methods

Methods for Experiment 1: Paid Students and Citizen Scientists in Neureka

Procedure. Paid students were recruited through advertising at Fresher's week, societies, mailing lists, online and physical advertisements and paid €50 for participation. To assess changes in mental health treatment during the study, participants were asked weekly if in the past week they had: 1) seen a healthcare practitioner for their mental health (yes or no), 2) taken an antidepressant (yes or no), and 3) received talk/psychotherapy (yes or no). Participants in the paid student arm were paid €50 if they completed 90% of their assessments. They also completed one extra item each week: the number of college assignments they had in the past week (0 to 3+, where 3 indicated 3 or more assignments). Besides this additional item, the procedures for the two samples were identical. The Citizen Scientist arm was not paid for participation; they downloaded the public version of the app on the Apple App Store or Google Play Store and completed the assessments without supervision. For Citizen Scientists who attempted to complete Multi-Mood more than once, we included assessments from the most complete attempt. In March 2021, Neureka added a mood graph feature to provide visual feedback to users. In the final sample of N=194, N=126 (65%) had access to the mood graph.

Data Preparation. Positive items were first reverse coded to reflect negative affect, e.g., enthusiastic became 'not enthusiastic'. This was to standardise the directional interpretation of edges between nodes and overall network connectivity across networks that vary in proportion of positive vs negative nodes. EMA items were rated on a 7-point Likert scale. To ensure that there was sufficient variance for each network to be estimated, for every item in their time series, we added a small amount of random noise, -0.1 to + 0.1, to each EMA item. This meant that networks were estimable even if a subject had rated a single item the same (i.e., with little to no variance) in their time series.

Sensitivity Analyses. Contemporaneous ($r > 0.51$, $p < 0.001$) and directed ($r > 0.25$, $p < 0.002$) connectivity were positively associated with network stability among Paid Students and Citizen Scientists. To understand if network stability might therefore bias our connectivity results in the two Neureka samples, we repeated our key analyses in these datasets with each participant's network stability as a covariate. Data missingness was low among our final samples (**Table S1**), and so we did not impute missing data. To confirm that this decision did not affect our results, we repeated our analysis of connectivity and baseline

depression using imputed data. We did this using the *amelia* package, which performs multiple (n=5) imputations of missing data in a multivariate time-series and averages across them to produce an imputed timeseries.

Permutation Tests. To test if these distributions had greater correlations than expected by chance, we performed permutation tests. Specifically, in all 5-node network combinations, we randomised the timeseries of each participant's EMA data. We estimated personalised contemporaneous and directed networks based on this randomised data for each of the 4,368 network combinations and determined the correlation between (randomised) network connectivity and depression for each. We determined the statistical significance for each network by counting the number of values in the shuffled distribution that exceeded the observed value for a given true network, i.e., its p-value. We repeated this for all combinations of networks and then found the proportion of networks that reached $p<.05$. Finally, we tested if the valence of nodes in the networks affected the results by examining whether the number of negative (vs. positive) EMA items in a given network influenced the association between depression and network connectivity.

Network Stability Test. In order to estimate each network's stability, we successively dropped, within-individual, up to 85% of each participant's data in increments of 5% and calculated the within-individual correlation between network structure among the full dataset and the down sampled data. For each participant, we calculated the proportion of data that could be dropped to retain a correlation of 0.70 with their full timeseries, i.e., correlation stability coefficient (CS coefficient). Based on previously recommended guidelines(1), the correlation stability coefficient should not be below 0.25 and ideally above 0.50. After examining internal reliability, we tested the generalisability of the structure of these networks across datasets by correlating the edge strengths (25 edges for the directed network, and 10 for the contemporaneous network) across the independent samples.

Regularised Networks. Regularising individual networks prior to averaging them is in our opinion inappropriate, as it zero-inflates the data and removes the benefit obtained from averaging across many networks. However, for the interested reader, we repeated our main analyses using regularised individual networks, that are then averaged over to derive a mean network connectivity score per participant. We estimated 100 5-node personalised regularised networks using the *graphicalVAR* package (nLambda = 50, gamma = 0). We chose to estimate a random subset of 100 5-node networks, instead of the full 4,368 5-node networks, due to the computational intensity involved with estimating regularised networks. Like with non-regularised networks, we tested the association between mean regularised

contemporaneous and directed network connectivity and i) baseline depression and ii) 8-week depression variability.

Full-16 Node Network. As a final step, we decided to check whether the results from a single full 16-node network would be similar to those obtained from the average of all 4,368 5-node networks. In both samples, we estimated a single 16-node network once for each participant and then correlated the resultant network connectivity (contemporaneous and directed) with baseline depression and depression variance.

Observed Power from Experiment 1. A power analysis was completed in R using the pwr package with alpha=0.05, power=0.8. The observed associations between network connectivity and baseline depression were relatively small and varied across our two samples. Before carrying out Experiment 1, we estimated power for detecting associations between the connectivity of both contemporaneous and directed networks and mean and variance of depression. The average Pearson R between baseline depression and mean contemporaneous network connectivity across Paid Students and Citizen Scientists was r=.15 (i.e., mean PS: r=.07 and CS: r=.23). At an alpha of p=.05, power of 0.8, a sample of N=323 is required to detect this. The same average for directed network connectivity was r=.11 (i.e., mean PS: r=.03 and CS: r=.19). A sample of N=645 would be required to detect this effect. In Experiment 2, the available sample size of our external dataset (HNATD) was N=519 and we were thus powered to test for contemporaneous network associations only. The effect size for the observed association between network connectivity and SD of depression was much stronger in both samples and for both network types (contemporaneous and directed). The average Pearson R between SD of depression and mean contemporaneous network connectivity across Paid Students and Citizen Scientists was r=.345 (i.e., mean PS: r=.29 and CS: r=.40). At an alpha of p=.05, power of 0.8, a sample of N=63 is required to detect this. The same average for directed network connectivity was r=.26 (i.e., mean PS: r=.21 and CS: r=.31). At an alpha of p=.05, power of 0.8, a sample of N=113 is required to detect this. In Experiment 2, the sample size of the HNATD was therefore well-powered to detect associations between network connectivity and variance in depressive symptomology.

Methods for Experiment 2: Replication in a large and independent Citizen Science sample (HNATD)

Procedure. At baseline, participants completed the Quick Inventory of Depressive Symptoms (QIDS)(2). Participants rated their mood on a visual analogue scale between 0 (not at all) – 100 (very much). Among the 43 questions that participants answered, we selected a subset of 11 items (6 positive and 5 negative) from the Positive and Negative Affect Schedule (PANAS). Items selected were relaxed, energetic, anxious, enthusiastic, nervous, content, irritable, calm, dull, cheerful, and tired. There were 1,302 participants who completed at least 1 EMA assessment and 519 that completed at least 75% of assessments and were analysed here (**Table S1**). We retained the 'gloomy' for a separate analysis described below.

Data Preparation. As with the Experiment 1 Neureka datasets, positive items were first reverse coded to reflect negative affect, e.g., enthusiastic became 'not enthusiastic'. The HNATD study used VAS scales from 0-100 and so variance in responding was not an issue for network estimation. Nonetheless, we added the same noise to the data as in Experiment 1 for consistency. More importantly perhaps, HNATD implemented a different EMA schedule to the Neureka studies; they had 3 assessments per day, resulting in unequal intervals between assessments overnight. To avoid this, we removed all overnight lags in our analyses, so the directed network edges uniformly reflect the 6-hour lag of one emotion onto another (and itself).

As a final step, we evaluated two key assumptions VAR models which are that the underlying data is both stationary and homoscedastic. We tested the individual time-series in both samples for stationarity using the KPSS test and found the majority of data (65%) were stationary. We also tested each time-series for homoscedasticity, using the Breusch-Pagan test, and found the majority of data (72%) were homoscedastic.

Data Analysis. Analysis was carried out in an identical manner to that described in Experiment 1. Because we do not have weekly depression scores, we used the standard deviation of the EMA item 'gloomy' as a proxy for the variance in depression and used it to test if network connectivity was best explained by variance in negative emotion, rather than its point-estimated severity per se. Note that the 'gloomy' item was not used in any network estimation analysis. For permutation analyses, as with the Neureka samples, participants classified as outliers in terms of their network connectivity scores were excluded on a per-network basis. The mean sample size in each network was therefore N=515.3, min=512, max=516.

Methods for Experiment 3: Comparison of Clinical and Non-Clinical Network Structure and Stability

Procedure. Data were gathered from 45 patients with a primary diagnosis of MDD or GAD who completed EMA for 30 days prior to undergoing therapy(3). At baseline, participants completed the Hamilton Anxiety Rating Scale (HARS)(4) and the Hamilton Rating Scale for Depression (HRSD)(5). Participants were prompted to complete EMA assessments 4 times per day for a minimum of 30 days and rated items on a visual analogue scale from 0 (not at all) to 100 (as much as possible). For each assessment, participants answered 26 questions related to symptoms of MDD, GAD, positive affect, negative affect, rumination, behavioural avoidance, and reassurance seeking. We selected only the positive and negative affect items, and removed items that were either behavioural, e.g., procrastinated, or physical, e.g., experienced muscle fatigue. After removing these questions, we were left with 17 items including: energetic, enthusiastic, content, irritable, restless, worried, worthless or guilty, frightened or afraid, loss of interest or pleasure, angry, hopeless, positive, fatigued, difficulty concentrating, accepted or supported, threatened, and dwelled on the past (**Table S2**). The 'down or depressed' item was excluded from network estimation but was retained for a separate analysis detailed below.

Data Preparation. No exclusions were applied to this dataset, as all participants provided to us had completed the study requirement of 30 days with at least one assessment per day (**Table S1**). As with in Experiment 1 and 2, positive items were reverse coded, and we added a small amount of noise to the data to ensure networks were estimable. Similar to HNATD, we removed all overnight lags in our analyses so that all directed edges referred to a 4-hour interval of one emotion onto another.

Data Analysis. Analysis was carried out in an identical manner to that described in Experiment 1 and 2. Because only baseline depression scores were available, we used the standard deviation of the 'down or depressed' item over the entire time-series to approximate depression variance, as a proxy for the weekly depression scores available in the Neureka samples. We then estimated the correlation between network connectivity and the variance of 'down or depressed'.

For permutation analyses, participants classified as outliers in terms of their network connectivity scores were also excluded on a per-network basis. The mean sample size in each network was therefore N=44.9, min=44, max=45.

Table S1: Comparison of demographic characteristics between Paid Students, Citizen Scientists, HNATD, and a clinical sample.

	Paid Students	Citizen Scientists	HNATD	Clinical Sample
**Sample Characteristics**				
Sample Size	155	194	519	40
Age (SD)[Range]	23 (4.7) [18, 41]	49.5 (13.6) [18, 82]	40.3 (13.6) [17, 73]	34.1 (13.2) [18, 60]
Gender				
% Female	74.8% (116)	64.9% (126)	83% (433)	65% (26)
% Male	23.9% (37)	32% (62)	17% (88)	35% (13)
% Other	1.3% (2)	3.1% (6)	NA	NA
% Depressed (N)	54.2% (84)	32.5% (63)	51.3% (266)	95% (38)
**Study Design**				
Depression Questionnaire	CES-D 20	CES-D 20	QIDS	HAM-D
Frequency	2x per day	2x per day	3x per day	4x per day
# PA items	7	7	6	5
# NA items	9	9	6	13
# Unique 5-Node Networks	4368	4368	462	6188
Response Option	Likert (-3 to +3)	Likert (-3 to +3)	VAS (0-100)	VAS (0-100)
Required # Assessments	84	84	68	30*
**EMA Data**				
Mean Num. Assessments	104.3 (5.4)	97.6 (7.4)	77.6 (5.5)	115.5 (13.6)
Min. Num. Assessments	84	84	68	87
Max Num. Assessments	112	112	90	151
Mean % Complete	93.2% (4.8%)	87.1% (6.6)	86.2% (6.1)	NA

The “Other” gender category includes: non-binary, transgender male, transgender female, and did not disclose. Percent depressed refers to the percentage of a given sample on or above threshold for depression on the respective self-report instrucment used in that study. For neureka datasets that was ≥ 20 on the CES-D, for HNATD > 5 on the QIDS, and for the clinical sample was > 7 on the HAM-D.

*Participants in Fisher et al., (2017) were required to complete a fixed number of assessments, but were asked to complete at least 1 assessment for 30 days.

Table S2: Positive and negative affect items used to construct 5-node networks

Dataset	Positive Affect Items	Negative Affect Items
Paid students and Citizen Scientists	Enthusiastic, Cheerful, Strong, Happy, Energetic, Content, Talkative	Irritated, Down, Lonely, Anxious, Guilty, Indecisive, Restless, Agitated, Worried
HNATD	Relaxed, Energetic, Enthusiastic, Content, Calm, Cheerful	Gloomy, Anxious, Nervous, Irritable, Dull, Tired
Fisher	Energetic, Enthusiastic, Content, Positive, Accepted/Supported	Irritable, Restless, Worried, Worthless/Guilty, Frightened, Loss of interest/pleasure, Angry, Hopeless, Down/Depressed, Fatigued, Difficulty concentrating, Threatened/Judged/Intimated/Dwelled on Past

Supplementary Results

Control and Sensitivity Analyses **in Experiment 1**.

For the exemplar network, adjusting for network stability had no effect on the association between connectivity (contemporaneous and directed) and depression variability in either Paid Students or Citizen Scientists (**Table S3**).

Per-participant mean contemporaneous network connectivity remains a significant predictor of depression variance when EMA Mean is controlled for ($\beta > 0.29$, $p < 0.001$) [**Figure S2. "Contemporaneous (EMA Mean)"**], when EMA SD is controlled for ($\beta > 0.15$, $p < 0.05$) [**Figure S2. "Contemporaneous (EMA SD)"**], and when EMA AR is controlled for ($\beta > 0.23$, $p < 0.001$) [**Figure S2. "Contemporaneous (EMA AR)"**] in both datasets. For per-participant mean directed networks, the association holds when EMA mean is included in the model in both datasets ($\beta = 0.20$, $p <0.01$) [**Figure S2. "Directed (EMA Mean)"**]. When EMA SD is included in the directed model, the effects become non-significant in both samples ($\beta > 0.09$, $p > 0.05$) [**Figure S2. "Directed (EMA SD)"**]. Similarly, when AR is included in the directed model, the effects are also rendered non-significant ($\beta > 0.14$, $p > 0.05$) [**Figure S2. "Directed (EMA AR)"**] in both samples. It is worth noting however that in such a model, neither AR nor connectivity are significant due to high collinearity (r=.84) between them, so rather than AR being superior, they are highly overlapping metrics (AR = the self-loops of emotions in the network). Next, we evaluated the proportion of variance explained by the different types of models (**Table S6**). Per-participant mean contemporaneous network connectivity explained approximately 9% of variance in Paid Students (2nd best model) and 16% in Citizen Scientists (3rd best model). Compared to contemporaneous networks, directed network models performed worse in both samples. Directed network connectivity ranked as the worst performing model in Paid Students and the second worst performing model in Citizen Scientists, explaining no more than 10% of the variance in depression variability.

We chose to examine baseline depression rather than the mean over 8 weeks because only baseline data were available in HNATD and Fisher. When we repeat the analysis of Neureka data using mean depression instead of baseline, the results are near identical to those for baseline. Contemporaneous (r=.19, p<.01) (**Figure S3A)** and directed connectivity (r=.23, p<.01) (**Figure S3B**) are associated with mean depression in Citizen Scientists, but not in Paid Students (both p>.05).

We repeated our main analyses with regularised networks as a sensitivity analysis. Consistent with the main results, there was a significant association between per-participant mean regularised contemporaneous network connectivity and baseline depression in Citizen Scientists ($r = 0.16$, $p < 0.05$) but not Paid Students ($r = 0.07$, $p = 0.39$) (Figure S7A). However, there was no significant association between per-participant mean regularised directed network connectivity and baseline depression in either sample ($r = 0.02$, $p > 0.05$) (Figure S7B). Moving on to 8-week depression variability, there was a significant association between per-participant mean regularised contemporaneous network connectivity and depression variability in both Paid Students ($r = 0.32$, $p < 0.001$) and Citizen Scientists ($r = 0.38$, $p < 0.001$). While regularised directed network connectivity was significantly associated with depression variability in Citizen Scientists ($r = 0.16$, $p < 0.05$) but not Paid Students ($r = 0.11$, $p = 0.27$). For contemporaneous networks, the pattern of significant associations with depression (baseline and variability) was the same regardless of network type (i.e., regularised vs. non-regularised). As expected, the impact of regularisation was more prominent in directed networks where zero inflation was evident.

Lastly, we evaluated the similarity in results between a single 16-node network and the average of 4,368 networks. In both samples, for both contemporaneous and directed networks, there was a significant association between network connectivity and depression variance ($p < 0.05$) (**Table S5**). Baseline depression was significantly positively associated with contemporaneous network connectivity in citizen scientists ($\beta = 0.23$, $p = 0.001$), but not paid students (Beta = 0.08, $p = 0.33$) Unlike the association with average network connectivity from 4,368 5-node networks, baseline depression was not significantly associated with directed connectivity connected from the full 16-node network ($\beta = 0.05$, $p = 0.45$). Overall, the results between the two approaches are very similar, but we decided to retain the main analyses with the average of 4,368 5-node networks because it reduces collinearity among items. Since this could affect the interpretation of observed edges between nodes.

Table S3: Association between network connectivity in the exemplar 5-node network with SD depression (Paid Students & Citizen Scientists), SD gloomy (HNATD), and SD down or depressed (Clinical Sample).

Dataset	Network	Model	Beta (SE)	p-value
Paid Students	Contemporaneous	Unadjusted	0.15 (0.08)	0.06
		Adjusted	0.01 (0.10)	0.92

	Directed	Unadjusted	0.19 (0.08)	0.02
		Adjusted	0.19 (0.08)	0.02
Citizen Scientists	Contemporaneous	Unadjusted	0.37 (0.07)	< 0.001
		Adjusted	0.31 (0.08)	< 0.001
	Directed	Unadjusted	0.23 (0.07)	0.002
		Adjusted	0.22 (0.07)	0.004
HNATD	Contemporaneous	Unadjusted	0.27 (0.04)	< 0.001
		Adjusted	0.27 (0.04)	< 0.001
	Directed	Unadjusted	0.04 (0.04)	0.32
		Adjusted	0.04 (0.04)	0.34
Clinical Sample	Contemporaneous	Unadjusted	-0.04 (0.15)	0.77
		Adjusted	-0.04 (0.16)	0.81
	Directed	Unadjusted	0.10 (0.15)	0.50
		Adjusted	0.10 (0.15)	0.53

In the unadjusted model, symptom variability ~ network connectivity, while the adjusted model individual network stability is added as a covariate, i.e., symptom variability ~ network connectivity + network stability. Overall, adjusting for network stability had minimal effect on the relationship between network connectivity and symptom variability.

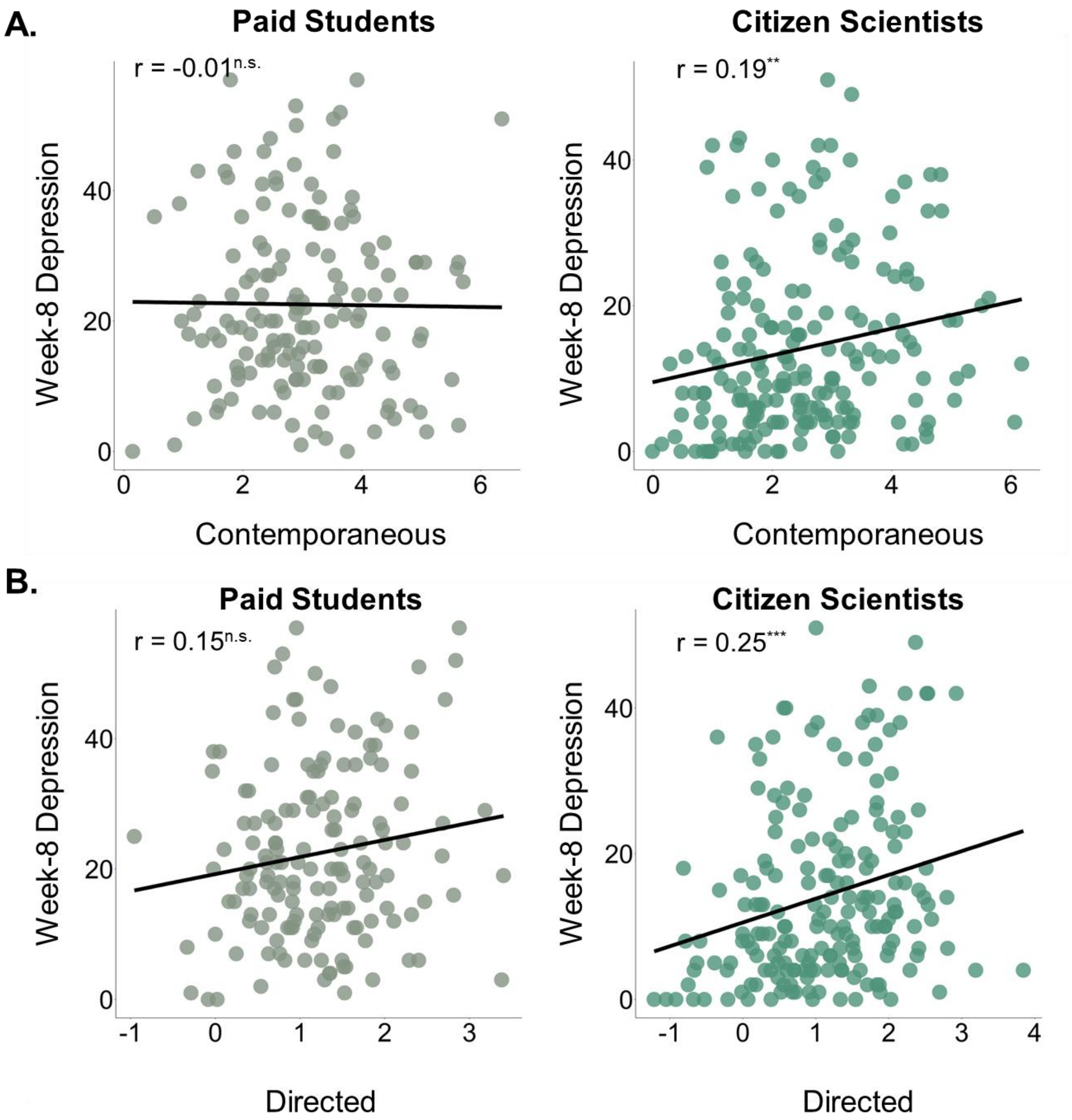

Figure S1: Association of per-participant network connectivity and Week-8 depression.

A) Contemporaneous network connectivity. Paid Students did not show a significant association ($r = -0.01$, $p = 0.89$) with Week-8 depression, but there was a significant association ($r = 0.19$, $p = 0.007$) for Citizen Scientists. B) Directed network connectivity. Citizen Scientist network connectivity was significantly associated with Week-8 depression ($r = 0.25$, $p < 0.001$), with no significant association for Paid Students ($r = 0.15$, $p = 0.06$).

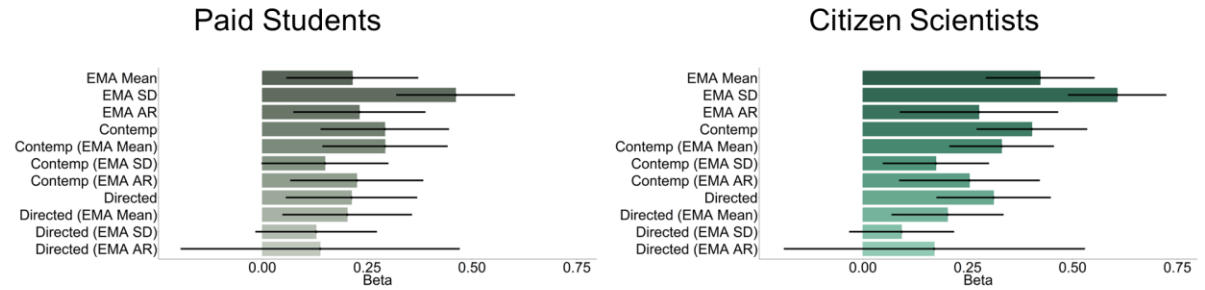

Figure S2: Comparative analysis of strength of association (x-axis, beta) between network connectivity and 8-week depression variance and other metrics derived from EMA (Mean, Standard Deviation [SD] and autoregression [AR]).

Contemporaneous connectivity remains a significant predictor of depression variance after controlling for mean, SD and AR. Directed connectivity only survives controlling for mean, but not SD and AR.

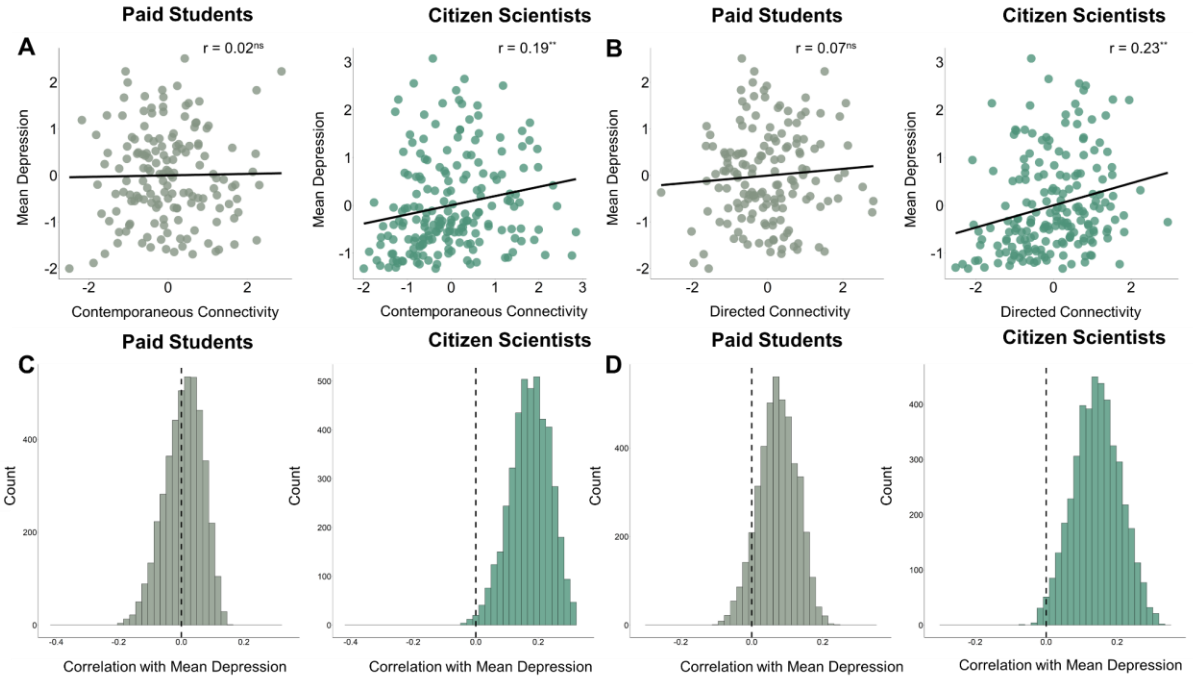

Figure S3. Replication of results using ‘mean depression’ instead of baseline depression.

A) Significant association between mean depression and per-participant mean contemporaneous network connectivity in Citizen Scientists but not Paid Students, B) Like with contemporaneous networks, there was a significant association between mean depression and per-participant mean directed network connectivity only in Citizen Scientists,

C) Histograms of association between contemporaneous network connectivity and mean depression for all 5-node networks in Paid Students and Citizen Scientists, D) Histograms of association between directed network connectivity and mean depression for all 5-node networks in Paid Students and Citizen Scientists.

Table S4: Association between depression variability and network connectivity either unadjusted or adjusted for mean EMA item variance.

Dataset	Network	Model	Beta (SE)	p-value
Paid Students	Contemporaneous	Unadjusted	0.29	<0.001
		Adjusted	0.17	0.05
	Directed	Unadjusted	0.21	0.008
		Adjusted	0.16	0.08
Citizen Scientists	Contemporaneous	Unadjusted	0.40	<0.001
		Adjusted	0.21	0.007
	Directed	Unadjusted	0.31	<0.001
		Adjusted	0.12	0.15
HNATD	Contemporaneous	Unadjusted	0.27	<0.001
		Adjusted	0.16	0.003
	Directed	Unadjusted	0.07	0.13
		Adjusted	0.12	0.04

Table S5: Results from full 16-node network are similar to the average of the 4,368 5-node networks.

16-node network connectivity was significantly associated with both baseline depression and SD depression in all cases as the average of the 4,368 5-node networks, with one exception. There was no significant association between directed network connectivity and baseline depression in Citizen Scientists ($\beta = 0.05$, $p = 0.45$).

Dataset	Network	Depression	Beta (SE)	p-value
Paid Students	Contemporaneous	Baseline	0.08 (0.09)	0.33
		SD	0.30 (0.08)	<0.001
	Directed	Baseline	-0.05 (0.09)	0.53

		SD	0.19 (0.08)	0.02
Citizen Scientists	Contemporaneous	Baseline	0.23 (0.07)	0.001
		SD	0.41 (0.07)	<0.001
	Directed	Baseline	0.05 (0.07)	0.45
		SD	0.16 (0.07)	0.03

Table S6: Proportion of variance explained for models predicting depression variance in Paid Students and Citizen Scientists.

Independent Measure	Paid Students		Citizen Scientists	
	Beta (SE)	R^2	Beta (SE)	R^2
EMA Mean	0.21 (0.08)	0.046	0.42 (0.07)	0.179
EMA SD	0.46 (0.07)	0.214	0.61 (0.06)	0.354
EMA AR	0.23 (0.08)	0.054	0.28 (0.10)	0.058
Contemporaneous	0.29 (0.08)	0.086	0.40 (0.07)	0.161
Directed	0.21 (0.08)	0.045	0.31 (0.07)	0.097

Contemporaneous network connectivity explained the second and third highest proportions of variance in depression variability in Paid Students and Citizen Scientists respectively. Given the overlap between depression variability and EMA SD, it is unsurprising that the EMA SD model explained the greatest proportion of variance. Compared to contemporaneous network connectivity, but also other measures of affect variability, directed network connectivity was a comparatively weak predictor of depression variability. In Paid Students, the directed network connectivity model explained the least variance in depression variability. While it was second to last performing model in the Citizen Scientist sample.

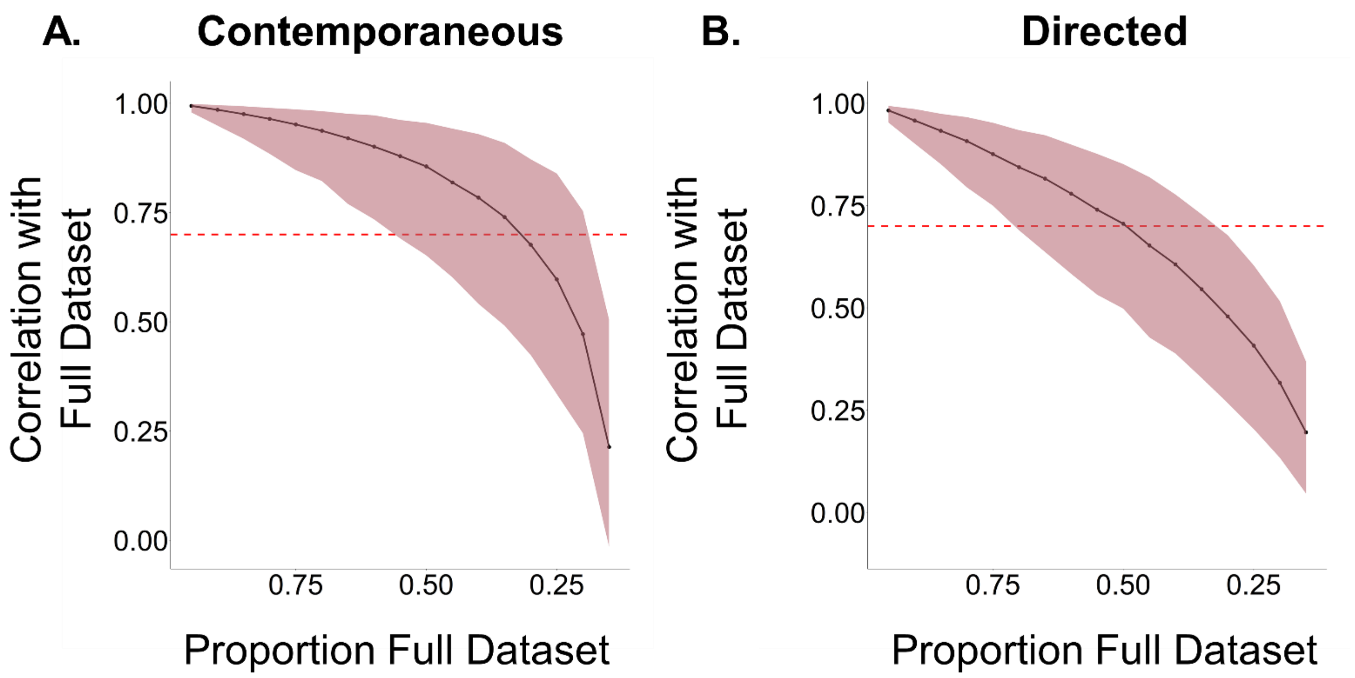

Figure S4: Exemplar network stability in a large independent sample (HNATD).

A) Contemporaneous exemplar network stability. The contemporaneous exemplar network had a correlation stability of 0.70. B) Directed exemplar network stability. The directed exemplar network had a correlation stability of 0.50.

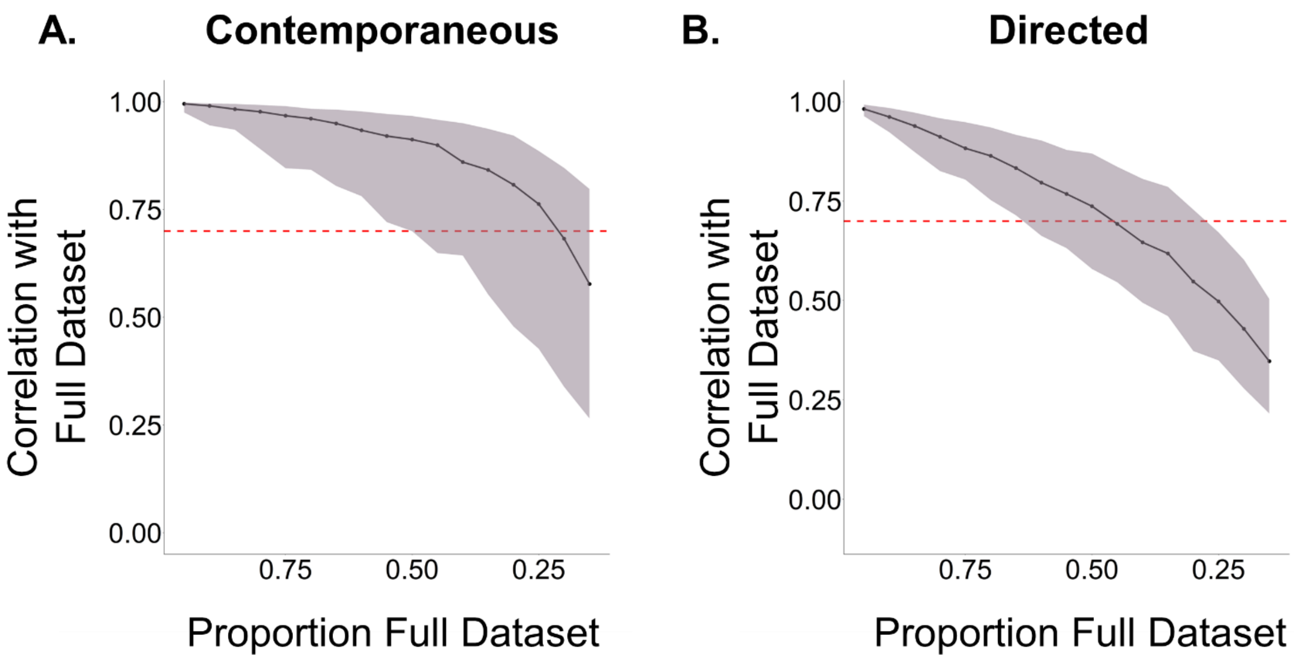

Figure S5: Exemplar network stability in a clinical sample

A) Contemporaneous exemplar network stability. The contemporaneous exemplar network had a correlation stability of 0.80. B) Directed exemplar network stability. The directed exemplar network had a correlation stability of 0.55.

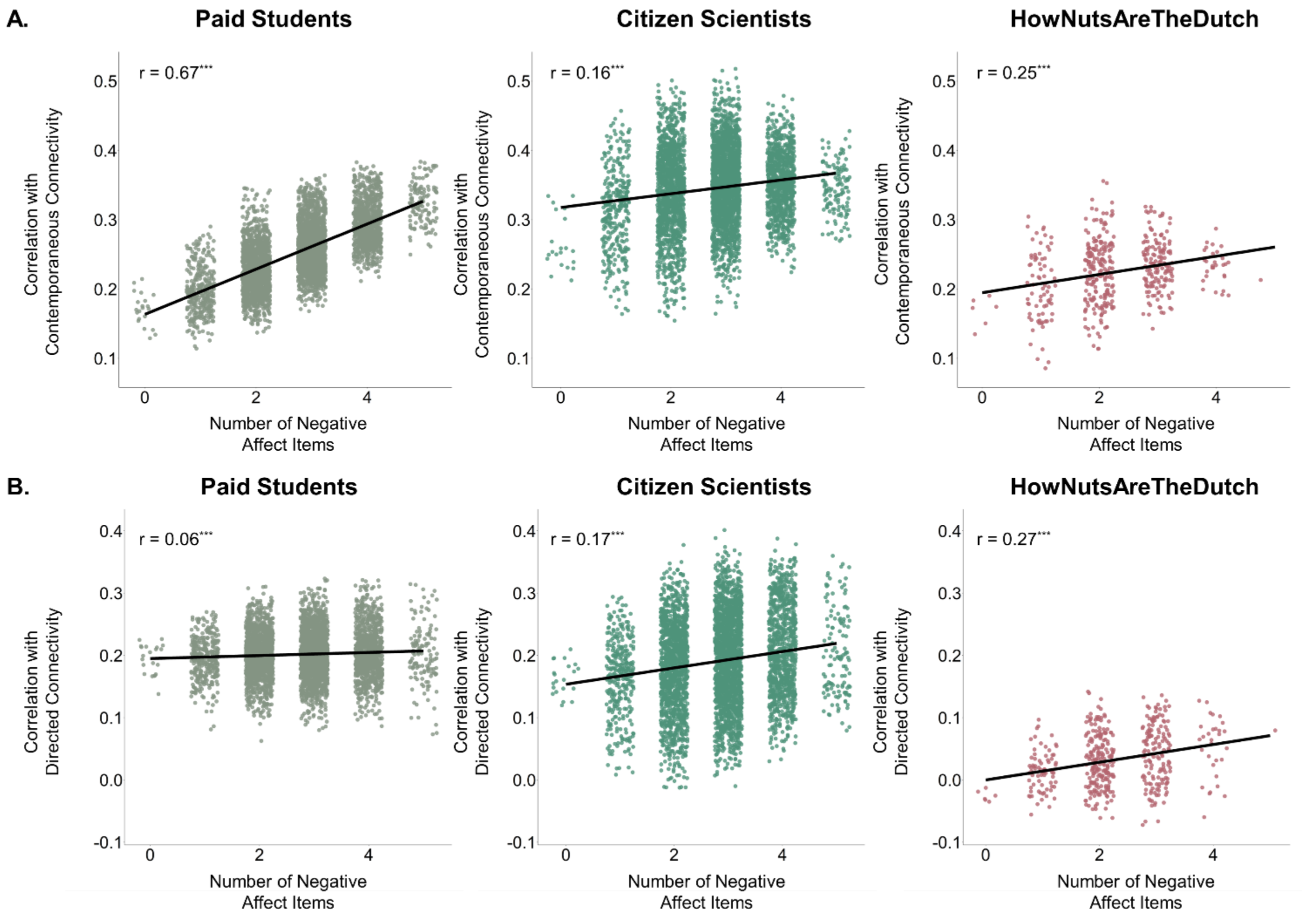

Figure S6: Impact of node valence on relationship between network connectivity and depression variability

Association between number of negative affect items and the correlation of depression variability and A) contemporaneous connectivity or B) directed connectivity. For Paid Students and Citizen Scientists, depression variability was operationalised as the SD of weekly depression scores. While in the HowNutsAreTheDutch sample, we used the SD of the 'I feel gloomy' item.

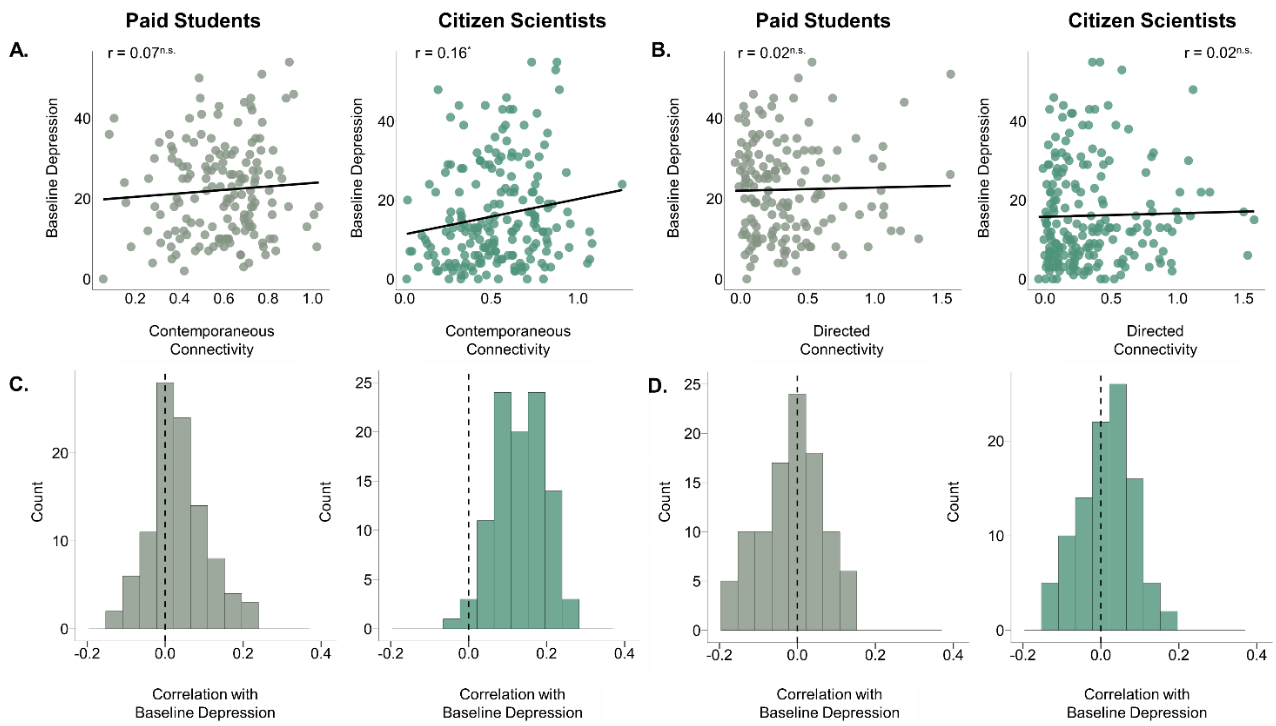

Figure S7. Regularised Contemporaneous and Directed Network Connectivity in Paid Students and Citizen Scientists.

A) Significant association between baseline depression and per-participant mean regularized contemporaneous network connectivity in Citizen Scientists but not Paid Students, B) There was no significant association between baseline depression and per-participant mean regularised directed network connectivity in either sample, C) Histograms of association between regularised contemporaneous network connectivity and baseline depression for 100 5-node networks in Paid Students and Citizen Scientists, D) Histograms of association between regularised directed network connectivity and baseline depression for 100 5-node networks in Paid Students and Citizen Scientists.

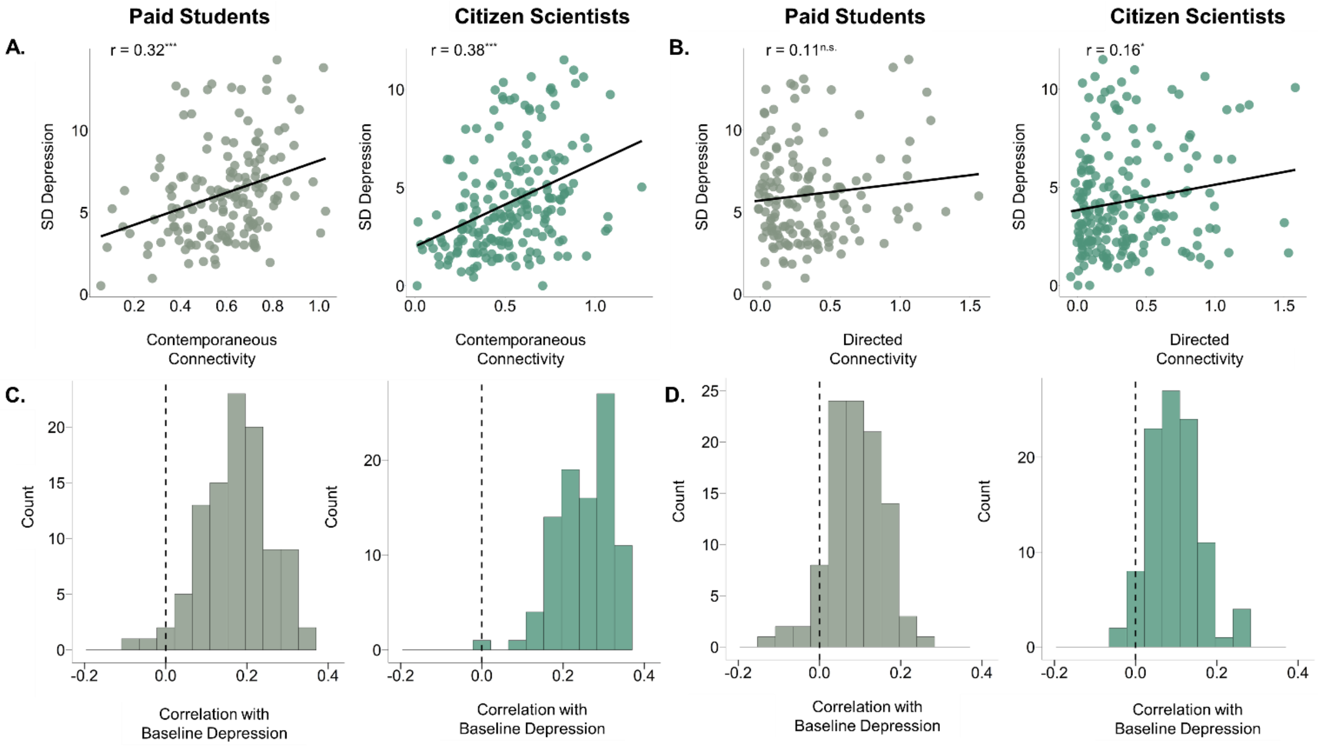

Figure S8. Depression variability is positively related to regularised network connectivity

A) Significant association between 8-week depression variability and per-participant mean regularized contemporaneous network connectivity in both Paid Students and Citizen Scientists, B) There was a significant association between 8-week depression variability and per-participant mean regularised directed network connectivity in Citizen Scientists but not Paid Students, C) Histograms of association between regularised contemporaneous network connectivity and 8-week depression variability for 100 5-node networks in Paid Students and Citizen Scientists, D) Histograms of association between regularised directed network connectivity and 8-week depression variability for 100 5-node networks in Paid Students and Citizen Scientists.

Supplementary References

1. S. Epskamp, D. Borsboom, E. I. Fried, Estimating psychological networks and their accuracy: A tutorial paper. *Behav Res Methods* **50**, 195-212 (2018).
2. A. J. Rush *et al.*, Comparison of self-report and clinician ratings on two inventories of depressive symptomatology. *Psychiatric Services* **57**, 829-837 (2006).
3. A. J. Fisher, J. W. Reeves, G. Lawyer, J. D. Medaglia, J. A. Rubel, Exploring the idiographic dynamics of mood and anxiety via network analysis. *Journal of abnormal psychology* **126**, 1044 (2017).
4. M. Hamilton, The assessment of anxiety states by rating. *British journal of medical psychology* (1959).
5. M. Hamilton, A rating scale for depression. *Journal of neurology, neurosurgery, and psychiatry* **23**, 56 (1960).
